# Process development for an effective COVID-19 vaccine candidate harboring recombinant SARS-CoV-2 delta plus receptor binding domain produced by *Pichia pastoris*

**DOI:** 10.1038/s41598-023-32021-9

**Published:** 2023-03-30

**Authors:** Sibel Kalyoncu, Semiramis Yilmaz, Ayca Zeybek Kuyucu, Dogu Sayili, Olcay Mert, Hakan Soyturk, Seyda Gullu, Huseyin Akinturk, Erhan Citak, Merve Arslan, Melda Guray Taskinarda, Ibrahim Oguzhan Tarman, Gizem Yilmazer Altun, Ceren Ozer, Ridvan Orkut, Aysegul Demirtas, Idil Tilmensagir, Umur Keles, Ceren Ulker, Gizem Aralan, Yavuz Mercan, Muge Ozkan, Hasan Onur Caglar, Gizem Arik, Mehmet Can Ucar, Muzaffer Yildirim, Tugce Canavar Yildirim, Dilara Karadag, Erhan Bal, Aybike Erdogan, Serif Senturk, Serdar Uzar, Hakan Enul, Cumhur Adiay, Fahriye Sarac, Arzu Tas Ekiz, Irem Abaci, Ozge Aksoy, Hivda Ulbegi Polat, Saban Tekin, Stefan Dimitrov, Aykut Ozkul, Gerhard Wingender, Ihsan Gursel, Mehmet Ozturk, Mehmet Inan

**Affiliations:** 1 Izmir Biomedicine and Genome Center, Izmir, Turkey; 2 Izmir International Biomedicine and Genome Institute, Dokuz Eylul University, Izmir, Turkey; 3 Pendik Veterinary Research and Control Institute, Istanbul, Turkey; 4 Marmara Research Center, TUBITAK, Kocaeli, Turkey; 5 University of Health Sciences, Istanbul, Turkey; 6 Ankara University, Ankara, Turkey; 7 Akdeniz University, Antalya, Turkey; 8 Present Address: VIB-UGent Center for Medical Biotechnology, Gent, Belgium; 9 Present Address: Lund University, Lund, Sweden; 10 Present Address: Erzurum Technical University, Erzurum, Turkey; 11 Present Address: Ankara Medipol University, Ankara, Turkey; 12 Present Address: Imperial College London, London, UK; 13 Present Address: Izmir Tinaztepe University, Izmir, Turkey

**Keywords:** Viral infection, Protein vaccines

## Abstract

Recombinant protein-based SARS-CoV-2 vaccines are needed to fill the vaccine equity gap. Because protein-subunit based vaccines are easier and cheaper to produce and do not require special storage/transportation conditions, they are suitable for low-/middle-income countries. Here, we report our vaccine development studies with the receptor binding domain of the SARS-CoV-2 Delta Plus strain (RBD-DP) which caused increased hospitalizations compared to other variants. First, we expressed RBD-DP in the *Pichia pastoris* yeast system and upscaled it to a 5-L fermenter for production. After three-step purification, we obtained RBD-DP with > 95% purity from a protein yield of > 1 g/L of supernatant. Several biophysical and biochemical characterizations were performed to confirm its identity, stability, and functionality. Then, it was formulated in different contents with Alum and CpG for mice immunization. After three doses of immunization, IgG titers from sera reached to > 10^6^ and most importantly it showed high T-cell responses which are required for an effective vaccine to prevent severe COVID-19 disease. A live neutralization test was performed with both the Wuhan strain (B.1.1.7) and Delta strain (B.1.617.2) and it showed high neutralization antibody content for both strains. A challenge study with SARS-CoV-2 infected K18-hACE2 transgenic mice showed good immunoprotective activity with no viruses in the lungs and no lung inflammation for all immunized mice.

## Introduction

The first report of coronavirus disease 2019 (COVID-19) was on December 2019^1^ and it nearly killed more than 6.5 million people worldwide as of 12 December 2022^2^. Although the first vaccinations started in late 2020, global vaccination rates are still low due to inadequate access in underdeveloped countries. Additionally, new variants of severe acute respiratory syndrome coronavirus 2 (SARS-CoV-2) are emerging and spreading where early-developed vaccines might not protect as expected^3^. The best way to prevent more transmissible and more dangerous variants from emerging is vaccine equity^4^. Of the first vaccines developed, Pfizer/BioNTech’s and Moderna’s mRNA vaccine require expensive manufacturing processes and ultra-low temperatures for storage/transportation. Cheaper and easier to handle vaccines are needed to better reach people in low- and middle-income countries.

There are currently more than 150 vaccines in different clinical stages^5^. Developed vaccines for COVID-19 can be mainly divided into two groups: (i) nucleic acid-based platforms including mRNA and viral vectors, and (ii) conventional vaccines such as attenuated/inactivated viruses, virus-like particles, and recombinant protein subunits. There is reduced durability of humoral immune responses for mRNA-based vaccines^6^. Although they induce high antibody titers at first, these usually wane after a few months^7^. On the other hand, recombinant protein-based vaccines are known to show durable immunoprotective effects with low side effects^8,9^. Recombinant vaccine production attracts attention for vaccination of low- and middle-income countries due to its effectiveness, affordable, and less demanding production processes^9,10^.

The SARS-CoV-2 virus uses its spike protein for cell entry. The receptor binding domain (RBD) of spike protein binds to angiotensin-converting enzyme 2 (ACE2), a cellular receptor, for its viral entry into the host cell. Therefore, the spike protein, especially its RBD, is an ideal candidate as a recombinant vaccine antigen to prevent the binding of the virus to ACE2. RBDs of other similar viruses, SARS-CoV^11,12^ and Middle East Respiratory Syndrome CoV (MERS-CoV)^13^, were previously developed as vaccine candidates.

Since the start of COVID-19 pandemic, several groups developed recombinant SARS-CoV-2 RBD-based vaccines and there are currently 16 recombinant spike protein-based vaccine candidates approved or in late clinical phases^8,10,14,15^. Most of these vaccines use the original SARS-CoV-2 strain that appeared in Wuhan in December 2019 but some of them was shown to be effective to other SARS-CoV-2 variants of concern^16–18^. In this study, we developed an effective vaccine candidate based on the SARS-CoV-2 Delta Plus variant (B.1.617.2.1). Delta and Delta Plus are highly contagious variants that appeared in India in late 2020 with three devastating waves, and it caused increased hospitalizations compared to other variants^3,19–21^. Delta Plus variant is the modified version of the delta variant (B.1.617.2) with an additional K417N mutation on its RBD.

The yeast *Pichia pastoris* (*Komagataella phaffii*) is an established microbial system for properly folded and post-translationally modified recombinant protein production. Besides therapeutics, it is used for the production of several established vaccines such as against Hepatitis B^22^ and influenza^23^. Here, the RBD of the Delta plus strain (RBD-DP) was cloned and expressed in *P.pastoris*. We produced the RBD-DP in 5-L bioreactors with a protein yield of more than 1 g/L of culture. After purification (> 95% purity) and performing the necessary in vitro characterizations, the RBD-DP was formulated with aluminum hydroxide (Alum) and CpG and tested for efficacy in vivo. The data demonstrated that our vaccine can stimulate effective virus-neutralizing antibodies, along with T-cell responses, and it effectively protects challenged mice from developing COVID-19 infection.

## Results

**Figure 1:**
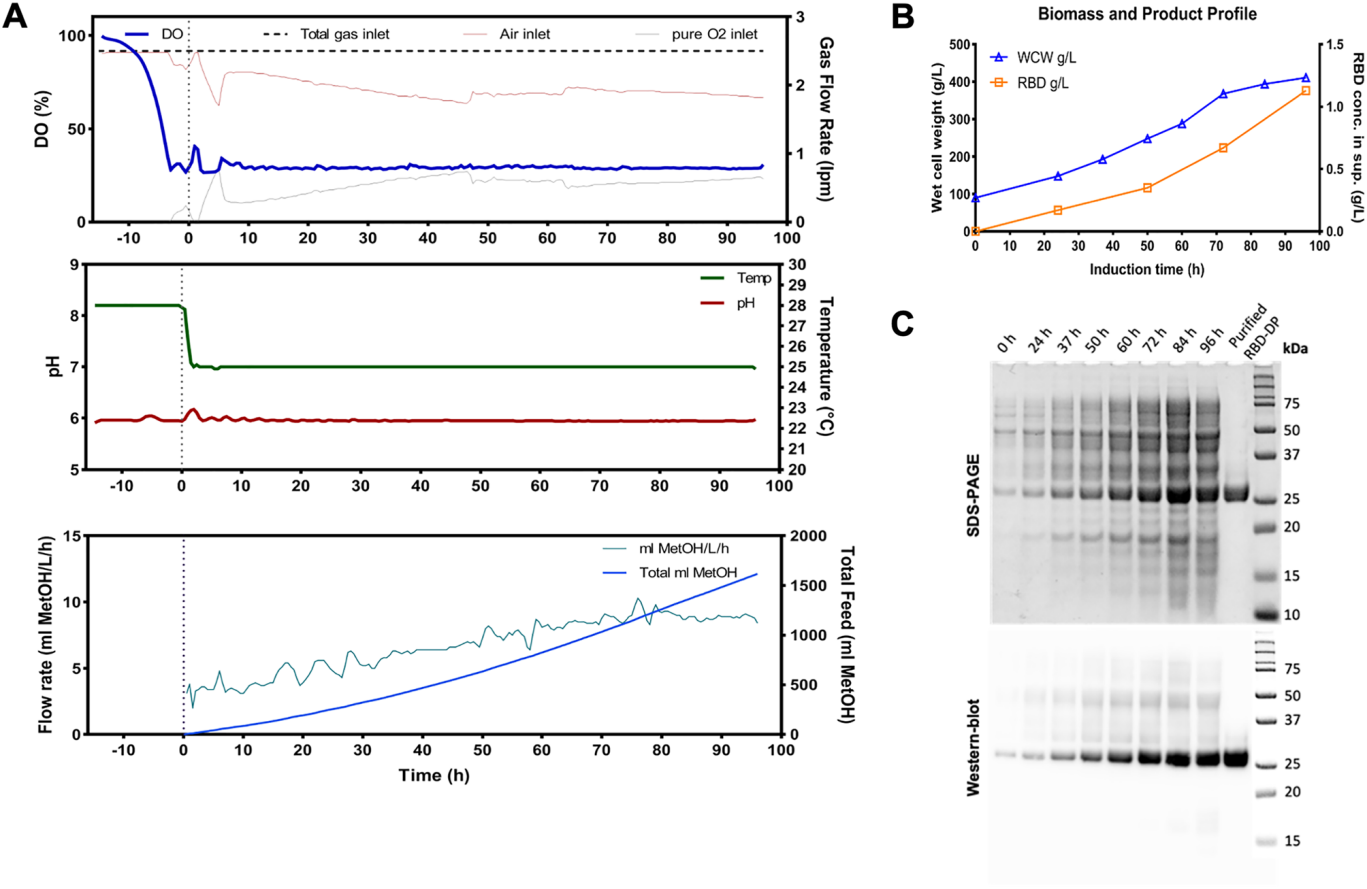
Fermentation process to produce RBD-DP at 5-L scale. (**A**) Course of dissolved oxygen tension (DO%) and ambient air/pure O_2_ sparger inlet (top panel), course of pH and temperature (middle panel), and course of total methanol (MetOH) added and feed flow rate (bottom panel) of the two phase fermentation. On the x-axis, the time point of induction is marked as 0 (zero). (**B**) Biomass (wet cell weight, WCW) and RBD-DP product formation during the induction phase. (**C**) SDS-PAGE and Western blot analysis of the time-course fermentation supernatants (RBD-DP appears just above 25 kDa marker).

For recombinant production, clones derived from *P. pastoris* transformed with the RBD-DP gene were screened for the best expression of the RBD-DP protein under the control of the *AOX1* promoter (Supplementary Fig. S1A,B). Two-phase fermentation was performed for RBD-DP production and the time course of the process parameters is shown in Fig. 1A. The first phase was a glycerol-fed-batch phase to increase the cell density before induction and the second phase was a methanol induction phase where the production of RBD-DP took place. RBD-DP production yield was quantified as 1.2 g/L of culture (Fig. 1B, Supplementary Fig. S1C). RBD-DP identity and its production over time were also confirmed by SDS-PAGE and western-blot analysis (Fig. 1C).

**Figure 2:**
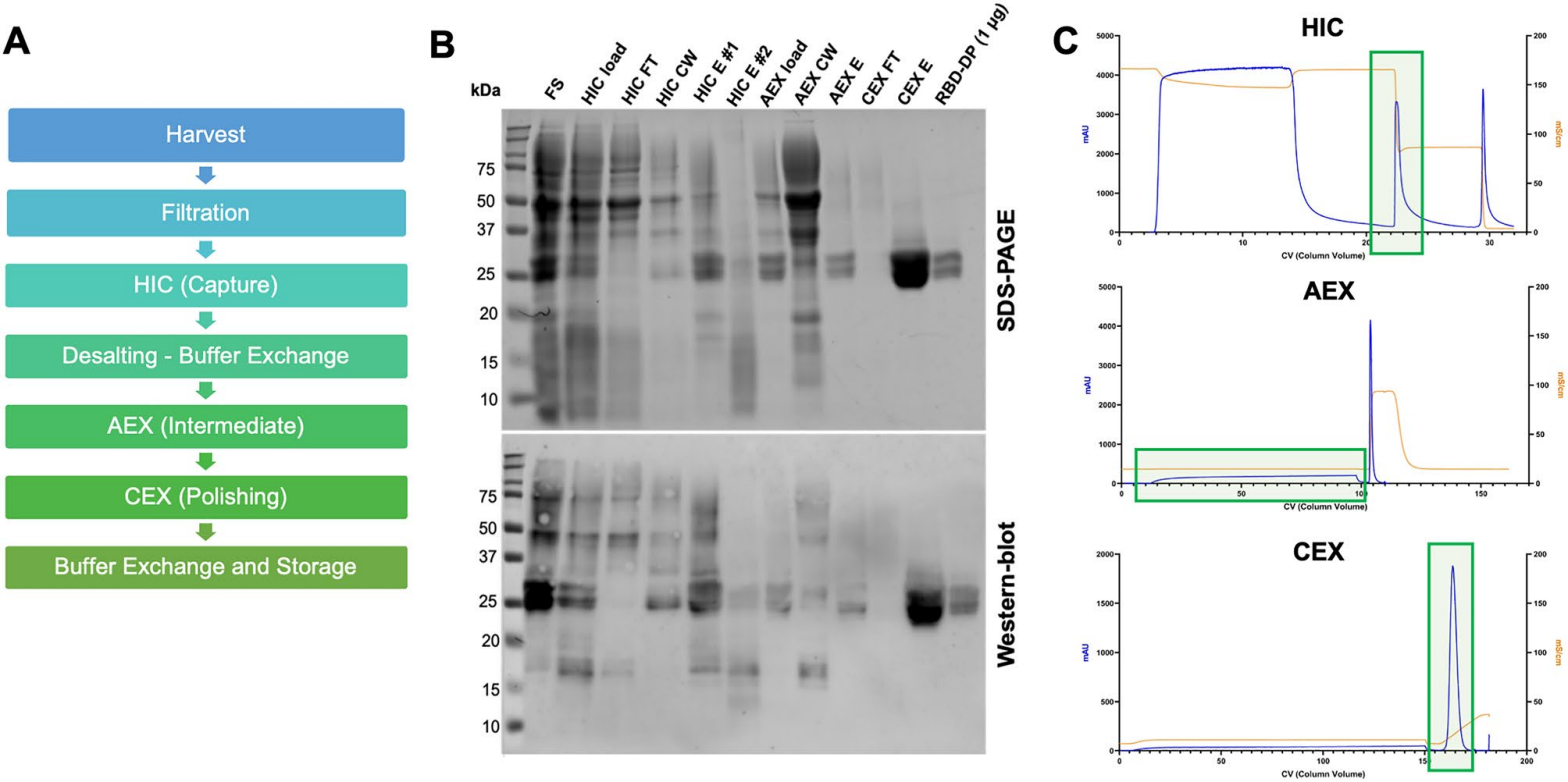
Purification of produced RBD-DP. (**A**) Purification flow-chart. (**B**) SDS-PAGE and Western blot analysis of the purification steps. *FS* fermentation supernatant, *HIC* hydrophobic interaction chromatography, *AEX* anion exchange chromatography, *CEX* cation exchange chromatography, *FT* flow-through, *CW* column wash, *E* elution. (**C**) Representative chromatograms of the purification process. Detection at 280 nm absorbance (mAU) and conductivity (mS/cm) were plotted. Fractions collected at each step are highlighted in light green boxes.

**Figure 3:**
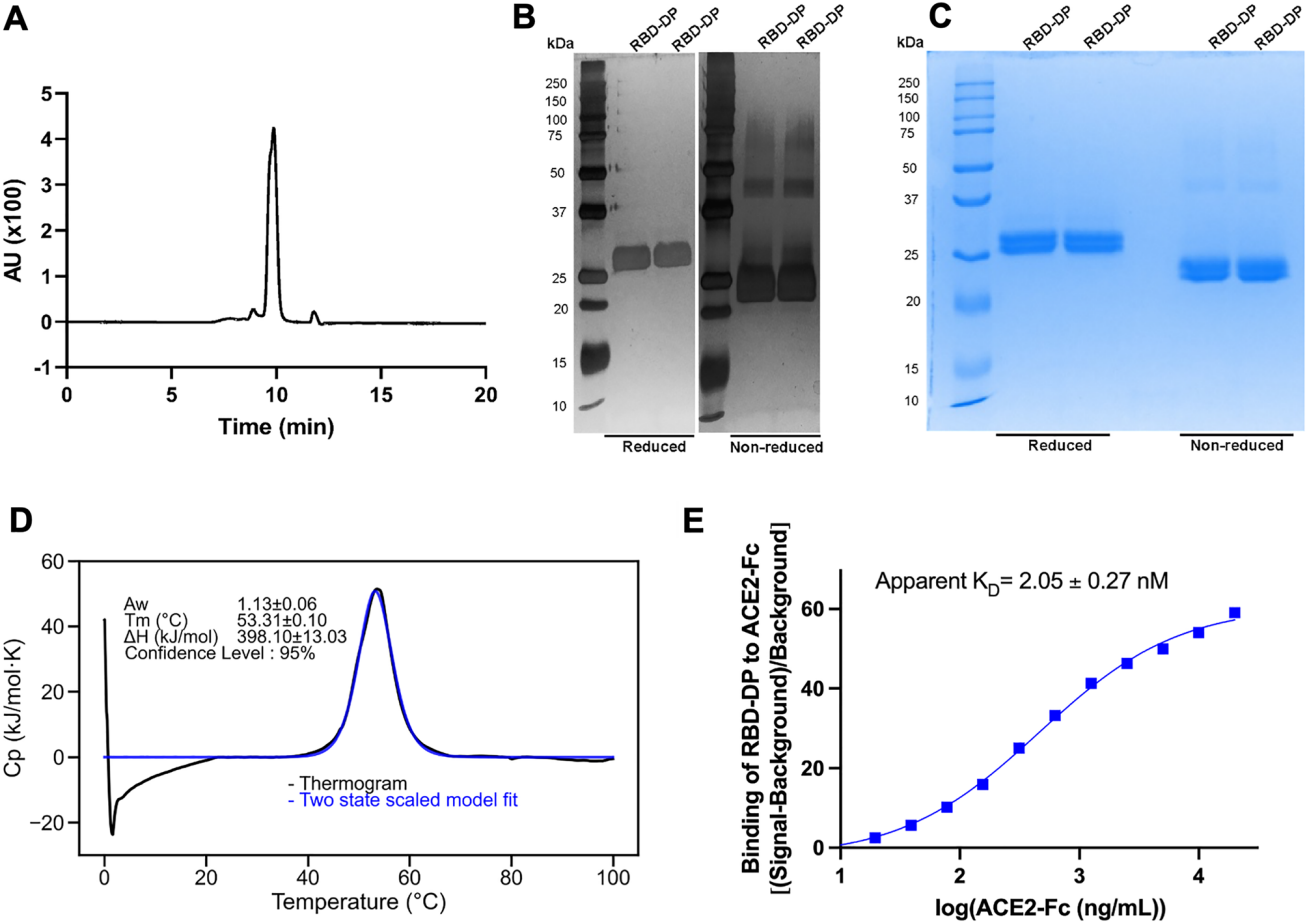
Characterization of the purified RBD-DP. (**A**) SE-UPLC chromatogram. (**B**) Silver stained SDS-PAGE of reduced/non-reduced RBD-DP. (**C**) Coomassie stained SDS-PAGE of reduced/non-reduced RBD-DP. (**D**) Differential Scanning Calorimetry (DSC) profile showing thermal melt of 53.31 ± 0.10 °C. (**E**) Determination of binding affinity of RBD-DP to ACE2-Fc by ELISA.

Since there were no added tags in the protein sequence, the multi-step purification process was optimized to isolate pure RBD-DP, which included hydrophobic interaction, anion exchange, and cation exchange chromatography steps (Fig. 2). The overall protein recovery was 20 ± 3% and its final purity was 96 ± 1% (Supplementary Table S1 and Fig. 3A). The identity of purified RBD-DP was confirmed by peptide mapping based on Quadrupole Time of Flight Mass Spectrometer (QTOF-MS) analysis, which gave 96% sequence coverage (Supplementary Fig. S2).

Purified RBD-DP was first characterized by Coomassie and silver-staining SDS-PAGE in reduced and non-reduced forms (Fig. 3B,C). The theoretical molecular weight of RBD-DP is 24.4 kDa and there is one N-glycosylation site at the amino acid position 12 (position 343 in SARS-CoV-2 spike protein). While the purified RBD-DP ran right above 25 kDa in its reduced form, the main band decreased to the right below 25 kDa (Fig. 3B). Highly glycosylated forms, appearing as a smeary higher band^24,25^, were more visible in the non-reduced gel images (Fig. 3B). To check if glycosylation affects RBD’s functionality or antigenicity, a Surface Plasmon Resonance (SPR)-based assay was performed on RBD-WT (Supplementary Fig. S3). Produced RBD-WT was first treated with Endoglycosidase H (EndoH), which deglycosylates N-linked glycoproteins. We confirmed that produced RBD-DP is highly N-glycosyalated (Supplementary Fig. S3A). The binding affinity of native glycosylated and deglycosylated RBD-WT to *in-house* ACE2-Fc was analyzed by SPR. There were no significant differences between the two both having affinities in low nanomolar range: K_D_ of 21 nM and 17 nM for glycosylated and de-glycosylated RBD-WT, respectively (Supplementary Fig. S3B,C).

The thermal stability of the produced RBD-DP was measured by differential scanning calorimetry (Fig. 3D). Its thermal melting point was 53.3 °C, which was slightly higher than that of RBD-WT (52.2 °C, Supplementary Fig. S3D). Furthermore, the binding affinity of RBD-DP to its native target, ACE2, was measured to have insight into its functionality and antigenicity. For this, the produced RBD-DP was immobilized onto the plate and different concentrations of *in-house* ACE2-Fc protein were added to get the binding curve. The apparent binding affinity of RBD-DP to ACE2-Fc was 2.05 nM (Fig. 3D), which is comparable to our SPR analysis of RBD-WT (Supplementary Fig. S3).

**Figure 4:**
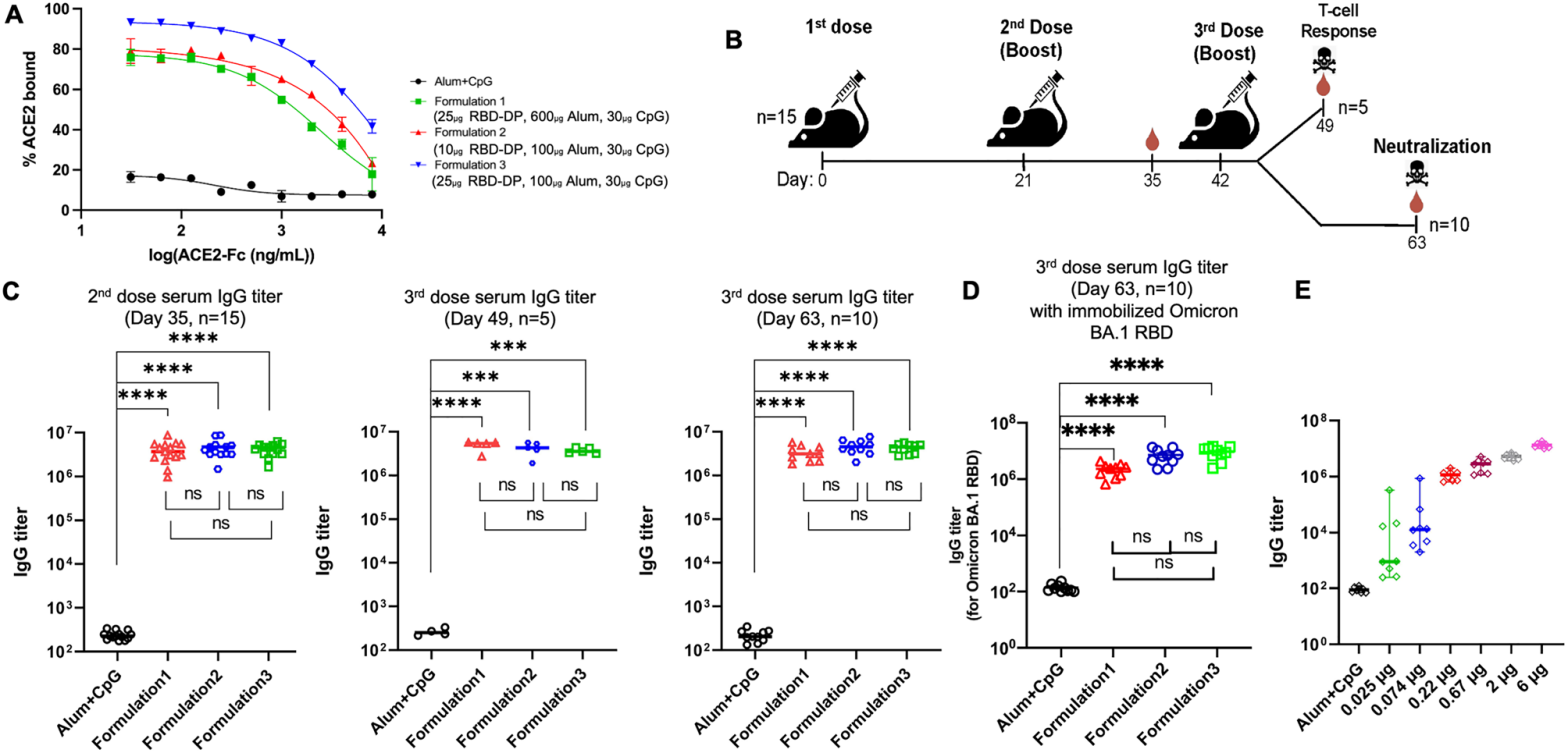
Humoral response induced by the RBD-DP vaccine. (**A**) ELISA-based ACE2 binding assay of RBD-DP in the three formulations used. (**B**) Study design for the animal immunizations. (**C**) Total IgG titers for the three different formulations after the 2nd and 3rd dose at days 35, 49, or 63. (**D**) Total IgG titer for Omicron BA.1 RBD for the serums after the 3rd dose at day 63. In ELISA set-up, instead of RBD-DP, the same amount of produced Omicron BA.1 RBD was immobilized on the plate. (**E**) Dose–response study with different amounts of RBD-DP (0–6 μg) in 100 µg Alum and 30 µg CpG. The total IgG titers reached plateau after 0.22 µg of RBD-DP. Formulation 1: 25 µg RBD-DP, 600 µg Alum, 30 µg CpG; Formulation 2: 10 µg RBD-DP, 100 µg Alum, 30 µg CpG; Formulation 3: 25 µg RBD-DP, 100 µg Alum, 30 µg CpG, Alum + CpG: 600 µg Alum, 30 µg CpG. Groups were compared by one-way ANOVA-Tukey’s multiple comparisons test (***p < 0.0005, ****p < 0.0001).

The produced and characterized RBD-DP was formulated with Alhydrogel (Alum) and CpG K3 in three different formulations. While the CpG concentration was kept constant at 30 µg, two different RBD-DP and Alum concentrations were tested: formulation 1 contained 25 µg RBD-DP and 600 µg Alum; formulation 2 contained 10 µg RBD-DP and 100 µg Alum, and formulation 3 contained 25 µg RBD-DP and 100 µg Alum. It is known that binding of RBD to ACE2 is the basic mechanism of SARS-CoV-2 neutralization. To test whether our formulated vaccine can still bind to ACE2, an ELISA-based assay was designed. Increasing concentrations of *in-house* ACE2-Fc were added to the formulated RBD-DP. And, after incubation to reach binding equilibrium, unbound ACE2-Fc was detected from the supernatant. We saw that all three formulations gave a high-binding plateau confirming their high-binding capacity to ACE2 (Fig. 4A). Interestingly, we observed that formulation 1 had lower ACE2-binding compared to that of formulation 3. The only difference was their Alum content (formulation 1 has 600 μg Alum compared to 100 μg in formulation 3), so we speculated that excess Alum might partially shield RBD-DP from binding to ACE2.

For animal immunizations, all three formulations were injected into mice (n = 15) in three doses in 21-day intervals (Fig. 4B). One week after the 3rd dose, 5 mice were euthanized for the analysis of the T-cell response. The remaining mice (n = 10) were euthanized 21 days after the 3^rd^ dose for the analysis of the antibody titers. Sera of mice were analyzed two weeks after the 2nd dose, as well as one and two weeks after the 3rd dose to measure total IgG titers (Fig. 4C). All three formulations gave high IgG titers between 10^6^ and 10^7^ after the 2nd and 3rd doses. Because omicron variants are currently in circulation, and we are in the process of producing Omicron BA.1 RBD domain (amino acid positions 332–550), we decided to test immunogenicity of our RBD-DP vaccine candidate against Omicron BA.1 variant (Fig. 4D). The sera from animals after 3rd dose (Day 63) were tested against immobilized Omicron BA.1 RBD protein (Supplementary Fig. S3E) to measure IgG titers. All formulations gave very high immunogenicity (IgG titer of ~ 10^7^, Fig. 4D).

To check the RBD-DP dose response, we designed another immunization study with 8 mice in each group. As in Formulations 2 and 3, 100 µg Alum and 30 µg CpG were used along with different doses of RBD-DP. With 0.22 µg RBD-DP, IgG titers reach a plateau at around 10^6^—10^7^ (Fig. 4E). The sera of two mice from the low-dose group (0.025 μg), of two mice from the high-dose group (6 μg), and one mouse from the control group were also analyzed with an ELISA assay using full-length spike protein (Supplementary Fig. S4). RBD-DP and full-length spike protein gave very similar signals confirming the high antigenicity of our produced RBD-DP.

**Figure 5:**
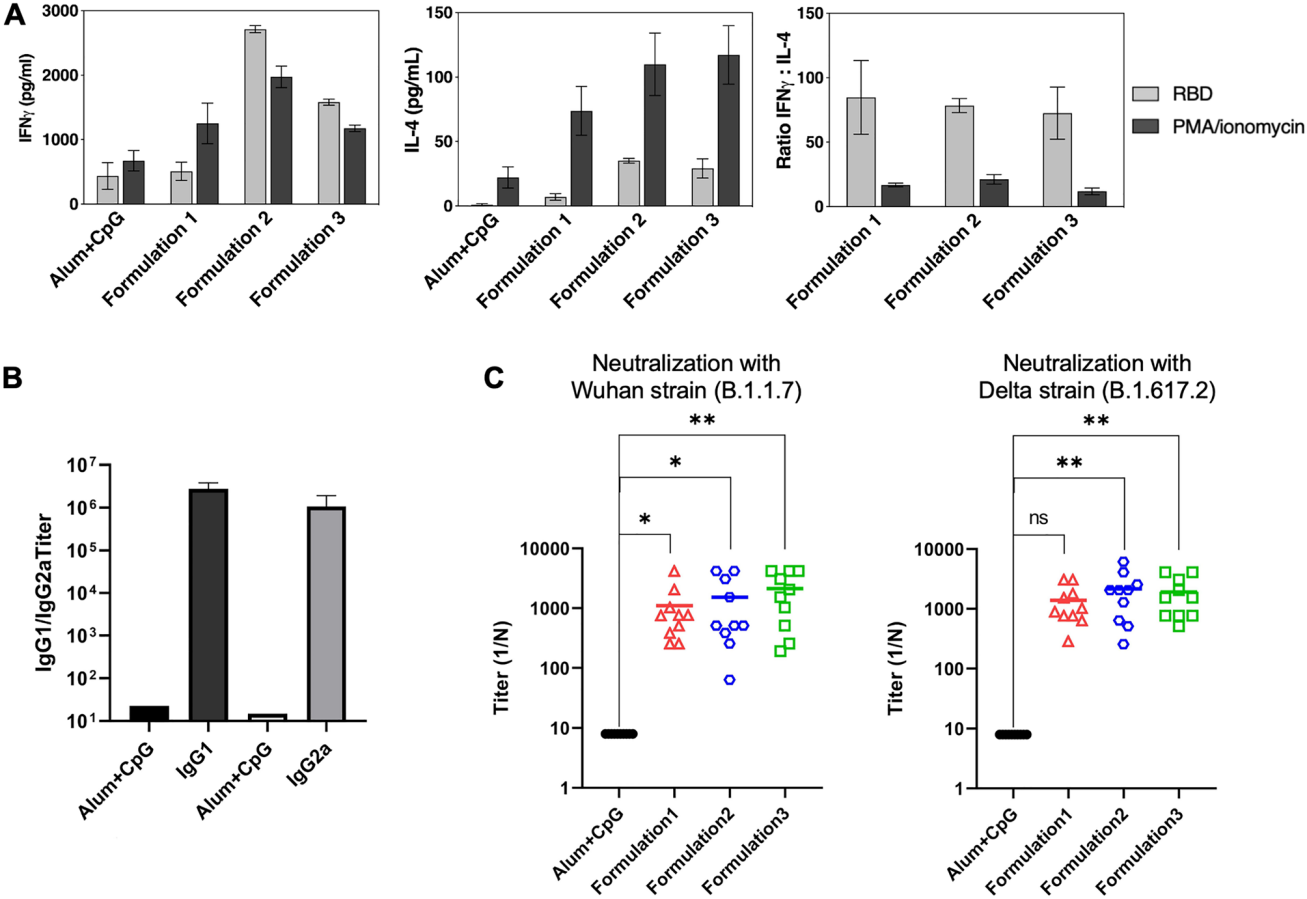
T cell response, IgG subtype and neutralization studies for the RBD-DP vaccine. (**A**) Splenocytes from mice (n = 5) of the indicated groups were stimulated with either RBD-DP (RBD, 24 h) or PMA/ionomycin (4 h) and the cytokine concentration in the supernatant was determined by ELISA. IFNγ (left panel) and IL-4 (middle panel) concentration and the IFNγ/IL-4 ratio (right panel). Representative data from at least two independent experiments are shown. (**B**) Titers of IgG1 and IgG2a subtypes in mice after immunized with formulation 2. (**C**) Live virus neutralization assay with two different strains of SARS-CoV-2 for mice immunized three doses with formulation 2. Formulation 1: 25 µg RBD-DP, 600 µg Alum, 30 µg CpG, Formulation 2: 10 µg RBD-DP, 100 µg Alum, 30 µg CpG, Formulation 3: 25 µg RBD-DP, 100 µg Alum, 30 µg CpG, Alum + CpG: 600 µg Alum, 30 µg CpG. Groups were compared by one-way ANOVA-Dunnett’s multiple comparisons test (ns: not significant, *p < 0.05, **p < 0.008).

One week after the 3rd dose (day 49), splenocytes from mice were isolated to measure the T-cell responses. To this end, splenocytes were stimulated either with RBD-DP (24 h) or PMA/ionomycin (4 h) and the concentration of the Th1 cytokine IFNγ and the Th2 cytokine IL-4 was determined by ELISA. All three vaccination formulations induced strong Th1 responses (Fig. 5A). When we checked the IgG subtype titers, we noticed a high induction of both IgG1 and IgG2a along with a balanced IgG2a/IgG1 ratio (Fig. 5B). Both IgG subtypes increase equally with increasing doses of RBD-DP (Supplementary Fig. S5).

Neutralizing antibody titers are highly predictive of immune protection and the RBD of Spike is targeted by many neutralizing antibodies^26^. High serum neutralizing antibody titers were obtained against both original Wuhan and Delta strains (Fig. 5C). Formulations 2 and 3 gave the highest neutralizing antibody titers (Fig. 5C). This suggests that lower Alum concentration might be better for the induction of neutralizing antibody response.

**Figure 6:**
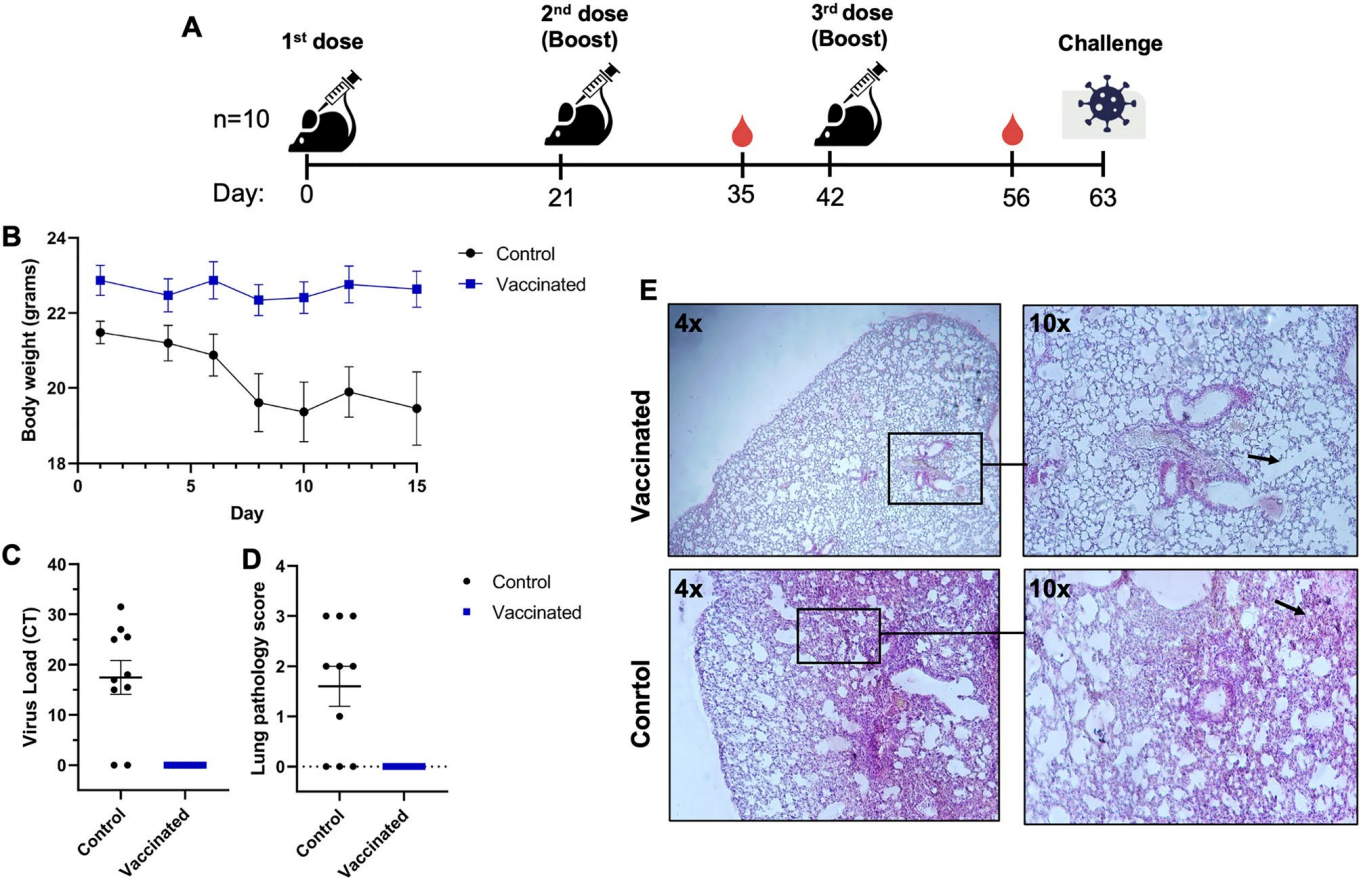
Immunoprotective activity in SARS-CoV-2-infected K18-hACE2 transgenic mice. (**A**) Study design for the animal immunizations and challenge. (**B**) Average animal weights post challenge. (**C**) CT (cycle threshold) values from qRT-PCRs against the nucleocapsid as indication of the infectious virus loads in lung homogenates by Real-Time PCR against the nucleocapsid as CT (cycle threshold) values. (**D**) Pathological inflammation scores of mice lungs. The lung inflammation was scored from 0 to 3 (0: no inflammation, 1: low, 2: medium, 3: high). (**E**) Representative images of hematoxylin–eosin stained lung tissues obtained from vaccinated (first row), and control (second row) groups with a magnification of 4 × (left) and 10 × (right).

Since we obtained strong neutralization responses (Fig. 5C), we next assessed the protection of immunized mice in a live virus challenge model. K18-hACE2 transgenic mice expressing human ACE2 were used for this assay^27,28^. Mice immunized with three doses of formulation 2 were protected from live SARS-CoV-2 challenge (Fig. 6). The control group lost 8–24% of their weight 8 days after infection (dpi), but there was no significant reduction in the mean weight of the vaccinated mice (Fig. 6B). At 10 dpi, 7 of the ten animals in the control group had significant weight loss (> 25%) and were euthanized in the final days of the experiment because three mice in this group were dying. Vaccinated animals completed the experiment without losing weight^29^. 12 days after the challenge, lung tissues of all animals were harvested at necropsy and analyzed. The virus load in the lungs of vaccinated animals was compared to those of control animals (Fig. 6C). The viral loads in the harvested lungs were determined using qRT-PCR with two primers that targeted the SARS-CoV-2 virus nucleocapsid gene^30,31^. Cycle threshold (CT) values from qPCR showed that 8 out of 10 animals in the control group had a high viral load (CT values > 15), which was consistent with the survival data. Viral load was not found in any vaccinated mice. When we checked the histopathology of their lungs, control animals had higher inflammation scores than vaccinated animals (Fig. 6D). Lungs of control group had interstitial inflammatory cell infiltration, alveolar septal thickening and more parenchymal infiltration at peribronchiolar regions compared to vaccinated animals which had relatively healthy lungs (Fig. 6E).

## Discussion

COVID-19 related mortality increases by age and vaccination is effective at preventing severe COVID-19 disease^32,33^. However, more affordable, and accessible vaccines are needed to fill the vaccine equity gap in low-/middle-income countries^34,35^. Here, we developed a recombinant protein subunit vaccine with the RBD of the SARS-CoV-2 Delta Plus strain (B.1.617.2.1).

The RBD of the Spike protein is a well-known target of many neutralizing antibodies, which is an essential protective mechanism against COVID-19^26,36^. Designing a recombinant protein subunit vaccine containing the RBD subunit of the Spike protein is a reasonable approach for vaccine development, but it needs an adjuvant to increase its immunogenicity^37–39^. Here, we used both Alum (Alhydrogel) and CpG K3 to reach high immunogenicity. In our initial tests, Alum alone as adjuvant did not induce sufficient titers of neutralizing antibody (data not shown) and, therefore, CpG was added to augment the immune response. CpG is an oligodeoxynucleotide that triggers Toll-like Receptor 9 (TLR9)-mediated innate immune responses that support subsequent adaptive immune responses^40^. Alum/CpG combination as an adjuvant was known to, directly and indirectly, activate many types of immune cells^41^.

For our immunization studies, three different formulations were used. While CpG concentrations were kept constant at 30 μg^42^, Alum concentrations were varied with high (600 μg) and low limitations (100 μg)^43^. These different formulations gave similar total IgG titers (Fig. 4C). When the RBD-DP amount was kept constant (25 μg) and the alum quantity was increased (100 μg in formulations 2 and 3, 600 μg in formulation 1), no significant difference was observed. Similarly, different amounts of RBD-DP (10 μg in formulation 2, 25 μg in formulation 3), together with 100 μg alum, resulted in comparable total IgG titers. Therefore, the lowest RBD-DP and alum quantities (formulation 2) were chosen for the rest of the study. To determine the minimum amount of RBD-DP required to reach these high IgG titers, a separate dose–response study was performed, which showed that as little as 0.22 μg RBD-DP could lead to ~ 10^6^ IgG titers (Fig. 4D). Before focusing on RBD-DP due to its more severe COVID-19 symptoms, we first started producing RBDs of Wuhan and UK (Alpha, B.1.1.7) strains. We analyzed IgG titers for Wuhan and UK variants which were much close to those of RBD-DP (Supplementary Fig. S6).

When splenocytes from immunized mice were stimulated in vitro with RBD-DP, it was shown that our vaccine promoted a high Th1 (high IFNγ) and low Th2 (low IL-4) response (Fig. 5A). Addition of CpG to the formulation might also helped this strong Th1 response which is essential for protection against infection^44^. While Th1 induces the production of IgG2a, Th2 stimulates the expression of IgG1^45^. We showed that IgG1 and IgG2a titers are very similar for our vaccine showing a balanced cell mediated immunity (Fig. 5B).

Irrespective of the IgG titers, it is critical to induce neutralizing antibody titers to achieve immune protection. Most SARS-CoV-2 neutralizing antibodies target the RBD of the spike protein, indicating that the RBD is immunodominant, and there is a strong correlation between levels of neutralizing and RBD-binding antibodies^26,46^. Besides its use as an effective vaccine candidate, the RBD can also be targeted for serological diagnostic tests.

As we obtained a strong neutralization antibody response, we next assessed the protection of immunized mice in a live virus challenge model. K18-hACE2 transgenic mice expressing human ACE2 were used as they represent an ideal model to study the pathological basis of mild or lethal COVID-19^27,28^. All the vaccinated animals were healthy after the challenge, while the lungs of the control group animals were highly inflamed (Fig. 6).

Overall, our findings show that a recombinant RBD-DP protein formulated with Alum and CpG is a good vaccine candidate for COVID-19 disease with high neutralizing activity and immune protection. Two doses instead of three doses could be enough for the same immune protection. As new SARS-CoV-2 strains are emerging, cross-strain protective immunity should also be tested^47,48^. Additional booster doses with the new variant’s RBD such as those of Omicron variants’ could be needed for protection against such variants^49–51^. Right now, we are trying to develop a similar process for RBD of SARS-CoV-2 Omicron strain. We even tested immunogenicity of our RBD-DP vaccine candidate against Omicron BA.1 variant (Fig. 4D), and we showed that our vaccine candidate might also protect against Omicron variants by having high IgG titers against those newly emerging Omicron variants. However, more tests are needed to see its neutralization and protection against each of those variants.

Using the *P. pastoris* system for large-scale production has several advantages due to its low-cost and high-yield production, which could help to fill the vaccine equity gap in low-/medium-income countries^4,34,35,52^. Here, we produced SARS-CoV2 Delta Plus strain recombinant protein in *P. pastoris* with high protein yield (> 1 g/ liter) and low-cost compared to other technologies. Our vaccine candidate generated high IgG titers against Wuhan, Delta Plus and Omicron BA.1 strains and showed high neutralization and immunoprotective activity. Our developed process can be easily applicable to other recombinant protein-based vaccine technologies.

## Methods

### Cloning and expression

The gene sequence of the Delta Plus variant’s SARS-CoV-2 RBD protein (RBD-DP) was designed from the SARS-CoV-2/Wuhan-Hu-1 complete genome (NCBI Gene ID: 43740568, amino acid positions 332–550) by incorporating the K417N, L452R, T478K and additional C538A mutations^53^. The RBD-DP gene was codon optimized for *Pichia pastoris* and inserted at the XhoI/NotI restriction sites of the *P. pastoris* expression vector pPicZαA (Life Technologies) by GenScript. *P. pastoris* GS115 host cells were transformed with the pPICZa-RBD-DP expression construct according to the manufacturer’s instructions (Life Technologies). In brief, approximately 1 μg of *Pme*I (New England Biolabs) linearized plasmid was transformed into host cells that were made competent by the lithium acetate method^54^. The competent cells were pulsed in 2 mm electroporation cuvette at 1.9 kV and 5 ms in a Micropulser Electroporator (Bio-Rad) and 1 mL of ice-cold 1 M sorbitol was added immediately afterward. Transformed cells were selected on YSD agar (10 g/L yeast extract, 20 g/L soytone, 20 g/L dextrose) plates containing 100 mg/ml zeocin. The transformants were allowed to grow for 3 days at 28 °C. Ten colonies were selected and streaked separately on YSD plates for single colony isolation. Then, one colony from each plate was picked and inoculated into 3 ml YSD broth and incubated overnight at 28 °C and 225 rpm. Frozen stocks of each colony were prepared. Additionally, the colonies were further expanded overnight by inoculated 30 ml of buffered glycerol yeast extract (BMGY) medium (1% yeast extract, 2% soytone, 2% glycerol, 100 mM potassium phosphate buffer pH 6.0, 1.34% yeast nitrogen base, 4 × 10^–5^% Biotin) in 250 ml baffled flasks with a starting OD_600_ value of 0.1. After incubation in BMGY for 18 h at 28 °C and 225 rpm, cells were harvested (3000*g*, 15 min, RT) and resuspended in 30 ml BMMY medium (1% yeast extract, 2% soytone, 1% methanol, 100 mM potassium phosphate buffer pH 6.0, 1.34% YNB, 4 × 10^–5^% biotin) to induce the protein expression. The induction was sustained by the addition of 1% (v/v) methanol twice a day for 72 h at 25 °C and 225 rpm. Cells were then harvested by centrifugation (3000*g*, 15 min, 4 °C). The supernatants were filtered through a 0.45 µm PES syringe filter and analyzed for the best production of RBD-DP protein by SDS-PAGE and Western Blot.

### Research cell bank production

The best producing *P. pastoris-*RBD-DP clone was used to generate a research cell bank as described by Sinha et al.^55^. Briefly, in a 50 ml baffled flask, 5 ml YSD broth (10 g/L yeast extract, 20 g/L soytone, 20 g/L dextrose) was inoculated with an aliquot of the frozen stock of the respective clone. The flask was incubated at 28 °C in a shaking incubator (225 rpm) for 24 h. Then, in a 250 ml baffled flask, 30 ml of BMGY medium was inoculated with the overnight culture with an initial OD_600_ of 0.1. The culture continued (28 °C, 225 rpm) up to an OD_600_ of 18–22, when 50% glycerol was added into the flask to a final concentration of 25% glycerol (v/v). This glycerol stock culture was aliquoted (1 ml/cryotube) and stored at − 80 °C.

### Fed-batch fermentation

The fermentations were performed in a 5-L Biostat B (Sartorius Stedim Biotech) fermenter with a maximum working volume of four liters. For the inoculum culture, a 1-L baffled flask containing 200 ml of BMGY was inoculated with 1 ml of frozen stock from the research cell bank and expanded for about 19 h (28 °C, 225 rpm) until reaching an OD_600_ of 18–22 (125 ml inoculum culture was used for fermentation). All cultures were initiated with 2.5 L BMGY medium and completed to four liters. The process was executed basically in two phases: the first phase was a glycerol batch phase during which the cell growth was promoted in BMGY. When all the sources were consumed by the cells, which was indicated by a sharp increase in dissolved oxygen tension (DO%) in the medium, 10 × YES solution (10% yeast extract, 20% soytone) was added to the vessel. Then the second phase started, in which the cells were fed with 100% methanol and the production of the target protein took place. Fermentation was performed at the following parameters throughout the process: stirrer rate 800 rpm, aeration rate 1 vvm, pH 6.0 (controlled by the addition of 25% ammonium hydroxide), and dissolved oxygen (DO) 30% saturation (provided by mixing pure oxygen into the inlet air as needed, keeping the total air flow rate constant at 1 vvm). Temperature was 28 °C for batch phase and 25 °C for methanol induction phase. Batch phase generally lasted between 15 and 17 h. Following a sharp rise in DO% indicating the depletion of the carbon sources in batch medium, 250 ml 10 × YES solution was pumped aseptically into the vessel. Methanol feeding was started subsequently to induce the protein expression. Temperature was decreased from 28 to 25 °C in 2 h and the induction was maintained at 25 °C for 96 h. The feeding solution (100% methanol containing 12 ml/L PTM1) was added at a linearly increasing rate starting from 3 ml/L/h and to 8 ml/L/h in 72 h, then kept constant till the end. In total, about 1600 ml of methanol was added during the induction phase. PTM1 contained 6.0 g CuSO_4_.5H_2_O, 0.08 g NaI, 3.0 g MnSO_4_.H_2_O, 0.2 g Na_2_MoO_4_.2H_2_O, 0.02 g H_3_BO_3_, 0.5 g CoCl_2_, 20.0 g ZnCl_2_, 65.0 g FeSO_4_.7H_2_O, 0.2 g biotin, and 5.0 ml H_2_SO_4_ per liter. The methanol feeding was stopped after 96 h of induction and kept till the DO spike to ensure the depletion of methanol in the broth. The cells were harvested by centrifugation (15,000 rpm, 30 min, 4 °C). The supernatant was filtered through a 0.45 µm cellulose acetate vacuum filtration unit and stored till the purification. Samples were taken at the beginning and end of the batch phase and every 12 h during the methanol induction phase. At each sampling point, 15 ml culture was taken and aliquots of 1.5 ml transferred into pre-weighed centrifuge tubes in triplicate and centrifuged (6000*g*, 5 min, 4 °C). The supernatants were collected and filtered through a 0.45 µm PVDF syringe filter into new tubes for further analysis. For biomass determination, pellets were washed two times with distilled water and weighed. Cell concentration was expressed as the wet cell weight (WCW) in g/L. The concentration of RBD-DP protein in the culture supernatants was determined by densitometric gel quantification with Image Lab software version 6.1 (Bio-Rad, USA). The identity of the protein was verified by Western blot analysis.

### Purification

After harvest, fermentation supernatants were first filtered through a 0.45 µm polyethylene sulfone (PES) filter. The filtered supernatants were adjusted to pH 8.0, 25 mM Tris, and 1.1 M ammonium sulfate. As a first purification step, a Butyl Sepharose High Performance (HiScreen Butyl HP, Cytiva) column was used for Hydrophobic Interaction Chromatography (HIC). The column was initially equilibrated with buffer (25 mM Tris, 1.1 M ammonium sulfate, pH 8.0). After filtration through a 0.22 µm PES filter, 30 mL of the supernatants were loaded onto the column at 75 cm/h. The column was washed with 8 column volumes (CVs) of buffer (25 mM Tris, 1.1 M ammonium sulfate, pH 8.0) to remove loosely bound species. The proteins were eluted with 25 mM Tris, 0.44 M ammonium sulfate during step elution. HIC elution pools were buffer exchanged with 25 mM Tris, 100 mM NaCl, pH 7.5 using HiPrep 26/10 desalting prepacked columns (Cytiva). As a second purification step, HiTrap Sepharose Q XL prepacked columns (Cytiva) were used for Anion Exchange Chromatography (AEX). Desalted samples were loaded onto the column with 25 mM Tris, 100 mM NaCl, pH 7.5 at 120 cm/h. The flowthroughs were collected. As a final purification step, Toyoscreen MxTrp65M prepacked columns (Tosoh) were used for Cation Exchange Chromatography (CEX). The columns were equilibrated with 6 CVs of 20 mM sodium phosphate, 20 mM sodium citrate, pH 4.6. Filtered samples were loaded onto the column at 100 cm/h. Linear gradient elution was applied for 15 CV at 150 cm/h. The proteins were eluted with approx. 20 mM sodium phosphate, 20 mM sodium citrate, 300 mM NaCI, pH 7.8. Pooled proteins were buffer exchanged with ultra-centrifugal filters (Amicon 10 K MWCO) into PBS (20 mM sodium phosphate, 100 mM NaCl, pH 6.5) for further use.

### Production of ACE2-Fc

A DNA fragment for the amino acids 1–740 of the human ACE2 (NG_012575.3) was cloned into the pDAD2 vector containing the Fc-domain of human IgG1 (P01857). The vector was transfected into CHO DG44 cells via nucleofector. Transfected cells were transferred to selection medium (OptiCHO (Gibco) with 5 µg/mL puromycin (Invivogen)). Single cell studies were carried out from the pooled cells that grew in the selection medium. Selected clones were tested in fed batch. All supernatants were collected and purified via Protein A affinity chromatography (Cytivia). Affinity-purified ACE2-Fc was subjected to size-exclusion chromatography (SEC) using a HiLoad 16/600 Superdex 200 pg column (Cytiva) equilibrated with PBS (pH 7.4) for polishing. Recombinant protein separated by SDS-PAGE was detected by Coomassie Blue staining and western blot with an anti-human IgG (H + L)-horseradish peroxidase antibody (Life technology). Recombinant protein aliquots were stored in PBS at − 80 °C.

### SDS–Page and western blot analysis

Protein yield and purity were determined by 12% Tris–Glycine gel electrophoresis. For the reduced samples, 200 mM DTT and 2 × Laemmli buffer, and for non-reduced samples, only 2 × Laemmli buffer were added to the protein samples and they were incubated at 70 °C for 10 min. Electrophoresis was performed using a Mini-Protean Electrophoresis System (Bio-Rad) running at 60 V for 30 min and then at 90 V until the dye front reached the bottom of the gel. Following electrophoresis, proteins were visualized by either CBB R-250 staining or silver staining according to the manufacturer’s protocol and scanned using ChemiDoc MP (Bio-Rad). For Western-blot analysis, gels were transferred to PVDF membranes and blocked with 1% casein in 1 × PBS. The RBD-DP was identified with a primary anti-SARS-CoV-2 Spike RBD antibody (Rabbit PAb, SinoBiological, Cat no. HD15SE2802) and a secondary antibody (goat anti-rabbit IgG (H + L), Invitrogen, Cat no. G21234). The blots were developed using the ECL Prime Substrate System and scanned using ChemiDoc MP.

### Size exclusion-ultra high-performance liquid chromatography (SE-UPLC)

A Waters UPLC system (Acquity H-Class) and Waters SEC column (1.7 μm 4.6 × 300 mm) with Waters SEC guard column (1.7 μm 4.6 × 30 mm) was used to determine the purity of the samples. The column was equilibrated with buffer (100 mM sodium phosphate, pH 6.8) for 20 min at a flow rate of 0.3 ml/min at room temperature (RT). 10 µl of the samples were injected into the column and absorbance at 280 nm was recorded.

### Reverse phase-high-performance liquid chromatography (RP-HPLC)

Reverse phase HPLC was performed on Agilent 1260 Infinity II equipped with G5654A bio-inert pump and G7165A multiple-wavelength UV detector. Sample injection volume was 10 µL. Samples were injected onto Thermo Scientific BioBasic-4 RP column (4.6 × 250 mm, 5 µm) run at 1 mL/min at 30 °C with a linear gradient from 30 to 60% mobile phase B over 10 min (Mobile phase A: 0.1% TFA (v/v) in ultrapure water and Mobile phase B: 0.1% TFA (v/v) in acetonitrile). Chromatograms were plotted with UV absorbance at 280 nm.

### Deglycosylation

4 μg of RBD protein was denatured with 1× Glycoprotein Denaturing Buffer (0.5% SDS, 40 mM DTT) at 100 °C for 10 min in 10 ml reaction volume. Then, 2 ml of 10× GlycoBuffer3 (1× concentration is 50 mM sodium acetate, pH 6) and 0.5 ml of EndoHf (NEB P0703) were added and completed with dH_2_O. The reaction mix was incubated for 1 h at 37 °C and analyzed by SDS-PAGE and Western Blot. A control reaction was performed under the same conditions but without adding the EndoHf to the reaction mix. The apparent molecular weight of EndoHf is 70 kDa on the gel.

### Peptide mapping

Purified samples containing 100 µg of RBD-DP in phosphate buffer were subjected to buffer exchange with 50 mM ammonium bicarbonate buffer using a 3 K centrifugal filter unit (Millipore). Next, 1% Rapigest solution (Waters) was added and proteins were denaturated at 80 °C for 15 min. Following denaturation, proteins were reduced through addition of 250 mM DTT (20 min, 60 °C), then alkylated with 250 mM iodoacetamide (30 min, RT). The digestion started following addition of 2 µg trypsin to the protein sample, was incubation at 37 °C overnight, and terminated through addition of formic acid. Digested peptides were diluted in sample dilution buffer (99:1:0.1 Water:Acetonitrile:formic acid, v/v/v) prior to analysis. Chromatographic separations were performed on a Waters Acquity H-Class Bio-UPLC column equipped with TUV detector and the MS measurements were performed on a Waters Xevo G2 XS-QTOF-MS column. The tryptic-digested peptide mixture was separated on a Waters BEH-130 C18 reversed phase column (2.1 × 100 mm 1.7 µm) at 65 °C with the following gradient: 0 min, 1% B; 60 min, 42% B; 61 min, 80% B; 64 min, 80% B; 65 min, 1% B, and 85 min, 1% B at a flow rate of 0.2 ml/min. The mobile phase A was 0.1% formic acid in water, while mobile phase B was 0.1% formic acid in acetonitrile. Eluting peptides were first detected by TUV detector at 214 nm and then by QTOF-MS with MS and MSE acquisition modes to obtain precursor ions and fragments ions, respectively. Data acquisition and processing was performed by Waters UNIFI software (v1.9.2). Cysteine carbamidomethylation was set as fixed, while methionine oxidation and asparagine deamidation was set as variable modifications. The number of minimum ions for peptide confirmation was set as 2. The match tolerance for fragment ions was 15 ppm.

### ELISA binding assay

High binding 96-well plates (Corning) were coated with 100 μL of 2 μg/mL RBD-DP diluted in 0.1 M Sodium Carbonate/Bicarbonate buffer overnight in duplicates at 4 °C. The next day, the wells were blocked with 1% (w/v) BSA in PBS, 0.05% Tween (PBS-T). After four washes with PBS-T, 100 μL serially diluted ACE2-Fc (in-house, 9.75–20,000 ng/μl) was added to the wells. The plates were incubated at room RT for 2 h to allow ACE2-Fc to bind to RBD-DP. Plates were washed four times with PBS-T followed by the addition of 100 μL 1:2000 diluted HRP-conjugated anti-human IgG antibody (Life technologies) and incubated for 1 h at RT. After this binding step, the plates were washed four times with PBS-T and two times with ultra-pure water. Finally, 100 μL TMB substrate (Invitrogen) was added and incubated for 15 min in the dark. The reaction was terminated with 100 μL stop solution (Invitrogen) and the absorption was measured at 450 nm using a Synergy H1 microplate reader (BioTek). Binding signals were reported as the background subtracted signal divided by the background. Hill equation was used to fit RBD-DP:ACE2-Fc binding curve and apparent K_D_ was calculated based on this fit (GraphPad Software).

### Surface plasmon resonance (SPR)

Binding kinetics were determined using a Biacore T200 instrument (Cytivia). All experiments were performed in HBS-EP buffer, pH 7.4. 10 nM ACE2-Fc protein was captured onto a Protein A Chip (Cytiva) at a flow rate of 10 µl/min for ∼1 min. A series of solutions ranging from 0 to 300 nM glycosylated and de-glycosylated RBD proteins were subsequently injected at a flow rate of 30 µl/min onto the ACE2-Fc captured surface. Data were corrected by double-referencing against a control flow cell containing no ACE2-Fc capture and against the flow cell with buffer injection. Sensogram curves were analyzed using the BiaEval 3.0 manufacturer's software. The dissociation constant (*K*_D_), association rate constant (k_on_), and dissociation rate constant (k_off_) values were calculated by fitting the kinetic association and dissociation curves to a 1:1 binding model. Extracted data were plotted via in-house Python scripts.

### Differential scanning calorimetry (DSC)

The DSC analysis of the RBD-DP protein was performed with Nano DSC (TA Instruments) with 300 ml active capillary cell volume. Prior to analysis, the RBD samples (at 1.0 mg/mL) and its buffer (20 mM sodium phosphate, 100 mM NaCl, pH 6.5) were degassed in a Degassing Station (TA Instruments) under 25 inches Hg vacuum pressure for 15 min. Buffer and sample were scanned consecutively between 0 °C to 90 °C at a rate of 1 °C/min. The data were processed using NanoAnalyze software (TA Instruments) by correcting the thermograms by subtracting the buffer blank scan and normalizing values to the protein concentration. The transition curve was fitted by the TwoStateScaled model to obtain the melting temperature (T_m_) of the protein.

### Formulation

Alum (Alhydrogel 2%, Invivogen, Cat no. vac-alu-250) and CpG (CpG K3, Ajinomoto) were used as adjuvants. Three different formulations of RBD-DP protein were prepared along with the negative control for animal immunizations: (a) formulation 1 = 25 µg RBD-DP, 600 µg Alum, 30 µg CpG, (b) formulation 2 = 10 µg RBD-DP, 100 µg Alum, 30 µg CpG, (c) formulation 3 = 25 µg RBD-DP, 100 µg Alum, 30 µg CpG; and (4) negative control = 600 µg Alum, 30 µg CpG. Briefly, RBD-DP protein in PBS was mixed with Alum by dropwise Alum addition, then CpG was added into this protein-Alum mixture. The solution was incubated at RT for 2 h in a tube rotator at 10 rpm. The pH and osmolality were measured and high protein binding efficiency (> 90%) was confirmed by SDS-PAGE.

### Formulated RBD-DP binding to ACE2-Fc

Alum-CpG control and different formulations of RBD-DP (Formulation 1, 2, 3) were blocked overnight with 5% (w/v) BSA. After three washes with PBS-T, serially diluted ACE2-Fc (in-house, 31.25–8000 ng/μl) was added and the samples were incubated for 2 h at RT. Then, the formulations and Alum-CpG were spun down at 13,000*g* for 5 min. Free ACE-2-Fc, which did not bind to the RBD on the Alum-CpG, remained in the supernatant. The ACE-2-Fc content in the supernatant was quantified by ELISA. For this, 96-well high binding plates (Corning) were coated overnight with 200 ng/well of RBD-DP protein. After blocking with 0.1% (w/v) BSA and four washes with PBS-T, 100 µL of each supernatant sample or ACE2-Fc standards (31.25–8000 ng/μl) were added to the wells in duplicates. Plates were washed four times with PBS-T and 100 μL 1:2000 diluted HRP-conjugated anti-human IgG antibody (Life technologies) was added and incubated at RT for 1 h. Plates were washed four times with PBS-T and two times with dH_2_O before 100 µL TMB solution (Invitrogen) was added. The reaction was terminated with 100 μL stop solution (Invitrogen) and absorption readings were taken at 450 nm using a Synergy H1 microplate reader. The results were analyzed by considering ACE2-Fc standard signals as 100% binding.

### Animal immunization studies

Animals used in this study were provided by the Vivarium Facility of the Izmir Biomedicine and Genome Center (IBG, Izmir, Turkey). All animal procedures were approved by the Animal Experiments Local Ethical Committee (AELEC) of IBG, Izmir, Turkey in accordance with national laws and policies. All the methods were carried out in accordance with national policies and the study is reported in accordance with ARRIVE guidelines. All methods were carried out in accordance with the approved guidelines and regulations. Animals were housed in individually ventilated cages under 22 ± 2 °C ambient temperature, 55 ± 10% relative humidity and 12:12 h light:dark cycle. Animal handling and injections were performed under aseptic conditions. Animal colonies used in this study were screened for the presence of any pathogen described by FELASA (Federation of European Laboratory Animal Science Associations) and were shown free from Mouse Hepatitis Virus (MHV) and Sialodacryoadenitis Virus (SDAV), which are substrains of the Coronavirideae virus family. For efficacy studies, 6–8 weeks-old female BALB/c mice were randomly allocated into four dose groups (Control, Formulation 1, Formulation 2, Formulation 3), with 10 animals used for the neutralization and 5 animals for the T cell response studies. 1 ml Insulin syringes with 26G needles were used to deliver the vaccine candidates. 100 µl formulation was injected into the back hindlimb muscles, with each leg receiving 50 µl of the formulation. After the initial injection (d0), two booster injections were performed after every three weeks (d21, d42). One week after the final injection (d49), five animals from each group (four from the negative control) were sacrificed to isolate splenocytes for the T cell response studies. Three weeks after the final injection (d56), the remaining 10 animals of each group were sacrificed for the neutralization studies. In both procedures, animals were sacrificed via cardiac puncture-mediated exsanguination under deep isoflurane anesthesia. Blood samples were collected using clot activator tubes, kept at RT for 30–60 min, centrifuged (1500*g,* 15 min) and sera were aliquoted into microcentrifuge tubes and stored at − 80 °C until further use.

### ELISA for IgG titer calculations

High binding 96-well plates (Corning) were coated with 200 ng/well in-house recombinant RBD-DP or full-length Spike protein diluted in 0.1 M Sodium Carbonate/Bicarbonate buffer at 4 °C overnight. The plates were washed 3 times with PBS, 0.05% Tween (PBS-T) then blocked at RT for 1 h with 1% BSA in PBS-T. Mice sera were diluted to 1:50 then serially diluted fourfold with 1% BSA in PBS-T. After blocking, the plates were washed 3 times with PBS-T, then 100 µl/well diluted mice sera were added, and the plates were incubated at RT for 2 h. HRP-conjugated anti-mouse IgG (Cell Signaling, 1:2000 diluted in 0.1% BSA PBS-T) was added as 100 µl/well and the plates were incubated at RT for 1 h. The plates were washed for four times with PBS-T and two times with ultra-pure water. TMB substrate (Life Technologies) was added as 100 µl/well and incubated for 5 min in the dark at RT, then 100 µl/well ELISA Stop solution (Invitrogen) was added into each well to stop the reaction. The optical density was measured at 450 nm. For the IgG2a/IgG1 titer measurements, alkaline phosphatase (AP)-conjugated antibodies were used. AP-conjugated anti-mouse IgG1 (Southern Biotech) and IgG2a (Southern Biotech) diluted as 1:1000 in 0.1% BSA PBS-T was added as 50 µl/well and the plates were incubated at RT for 2 h. The plates were washed four times with PBS-T and then two times with ultra-pure water. For the development of the plates, 50 µl/well p-nitrophenyl phosphate (PNPP, ThermoFisher) substrate solution was added. The optical density was measured at 405 nm every 30 min for 3 h. IgG titers were calculated for each serum sample as follows: first, the absorbance of each serum was subtracted from the blank and divided by the blank and then, obtained result was multiplied by the dilution factor of 50 for the negative control, by the dilution factor of x (x is the dilution factor with the absorbance value close to 1.0) for the other groups. The results were analyzed using GraphPad Prism software using One-way ANOVA to evaluate the statistical differences.

### T-cell response studies

Spleens were homogenized by filtering through a 76 µm mesh with ice cold PBS (Lonza), washed twice, and the red blood cells were lysed with ACK lysis buffer (Lonza). Single-cell suspensions were resuspended in complete RPMI 1640 medium (Life Technologies); supplemented with 10% (v/v) fetal calf serum (FCS), 1% (v/v) Pen-Strep-Glutamine (10,000 U/ml penicillin, 10,000 μg/ml streptomycin, 29.2 mg/ml L-glutamine (Life Technologies)) and 50 μM β-mercaptoethanol (Sigma)). The cells were then stimulated in vitro with either RBD-DP (10 μg/ml) for 24 h or with PMA (50 ng/mL) and ionomycin (500 ng/mL) (both Sigma-Aldrich) for 4 h at 37 °C before the cell culture supernatants were collected. The IFNɣ and IL-4 cytokine levels in serum were measured with the respective Sandwich-ELISA kits (BioLegend) according to the manufacturer’s instructions. The colorimetric changes were measured as absorbance at 450 nm (OD_450_) with a Spectrophotometer (Thermo Scientific, Multiskan FC Microplate Photometer) and the titers were defined from the reciprocal value of the absorbance. Data are presented as mean ± standard error of the mean (SEM). The statistical analysis was performed with GraphPad Prism 7.0 software.

### Virus neutralization assay

A micro-neutralization assay was carried out to detect SARS-CoV-2 neutralizing antibodies^56^. Authentic Wuhan strain and Delta strain, which were isolated in the Pendik Veterinary Control Institute, were used in the neutralization test (GISIAD gene bank accession number for Wuhan: ID EPI_ISL_491476, virus Name: hCoV-19/Turkey/Pen07/2020, for Delta: ID EPI_ISL_14540166, virus name: hCoV-19/Turkey/PD02/2021). The tests were conducted in Vero cell cultures (2.5 × 10^5^ cell/ml) grown in 96-well microplates. Briefly, sera were heat-inactivated at 56 °C for 30 min. Two-fold serially diluted sera in duplicates (starting from 1:8) were mixed with an equal volume of 100 ‘tissue culture infectious dose’ (TCID_50_) of SARS-CoV-2 and incubated for 1 h at 37 °C. Each dilution was transferred in quadruplicates onto Vero cells. After 4 days of incubation at 37 °C with 5% CO_2_, plates were checked for cytopathic effects by an inverted optical microscope (Olympus, CKX41). Neutralization titers were defined as the reciprocal of the highest dilution of the sera which showed at least 50% neutralization and titers were calculated based on the “Kärber Calculation”^57^. Test were conducted in BSL-3 laboratory conditions.

### SARS-CoV-2 challenge studies

K18-ACE2 transgenic BALB/c mice (8–10 weeks old, 10 mice/group) were injected three times into the back hindlimb muscles (each leg receiving 50 µl of formulation) 21 days apart with formulation 2 (10 µg RBD-DP, 100 µg Alum, 30 µg CpG) and with 1× PBS (control group). On day 63 (21 days after 3rd injection), mice were challenged intranasally for three consecutive days with live SARS-CoV-2 virus (10^5^ TCID_50_ in 50 µl SARS CoV2; GISIAD gene bank, Accession number ID EPI_ISL_491476, Virus Name: hCoV-19/Turkey/Pen07/2020, Delta strain B.1.617.2) at TUBITAK MAM’s BSL3 Animal facility (Ethical Committee Approval No: 16563500-111-60, 07/04/2021). The study is reported in accordance with ARRIVE guidelines. Mice were monitored each day after challenge for symptoms and weight. 12 days after the last virus challenge, blood was collected from heart under anesthesia and mice were sacrificed. Their lungs were collected and divided into two for histopathology and virus extraction procedures. The viral load in the lung specimens was assessed by Real-Time PCR using primers specifically detecting SARS-CoV-2 nucleocapsid genes (NC1 and NC2). The viral RNA was extracted from samples with the QIAamp Viral RNA Mini kit (QIAGEN) according to the kit instructions. Viral RNA detection was performed using specific primers with One Step PrimeScript III RT-qPCR Kit (Takara). All PCR reactions were performed on a CFX96 Touch instrument (BioRad) with the following Real-Time PCR conditions: 52 °C for 5 min, 95 °C for 10 s, followed by 44 cycles at 95 °C for 5 s and 55 °C for 30 s. For histopathological analysis, lung samples were fixed in buffered 10% formaldehyde solution for 48–72 h. The tissues were dehydrated and 5 μm sections were obtained in a temperature-controlled paraffin station on a sliding microtome. The tissue sections were stained with Hematoxylin–Eosin. All sections were evaluated using a bright field microscope with a camera attachment. The lung inflammation was semi-quantitatively scored with a 0 to 3 scale (0: no inflammation, 1: low, 2: medium, 3: high).

## Supplementary Information

Supplementary Information 1.

Supplementary Information 2.

## Data Availability

The data that support the findings of this study are available on request from the corresponding author.
