# Supplementary material for: Process development for an effective COVID-19 vaccine candidate harboring recombinant SARS-CoV-2 delta plus receptor binding domain produced by Pichia pastoris
Source: Sci Rep. 2023 Mar 30;13:5224. doi: 10.1038/s41598-023-32021-9 (PMC10062263; doi:10.1038/s41598-023-32021-9)
Supplement: Supplementary file 1 — Supplementary Information 1. [file 41598_2023_32021_MOESM1_ESM.docx]

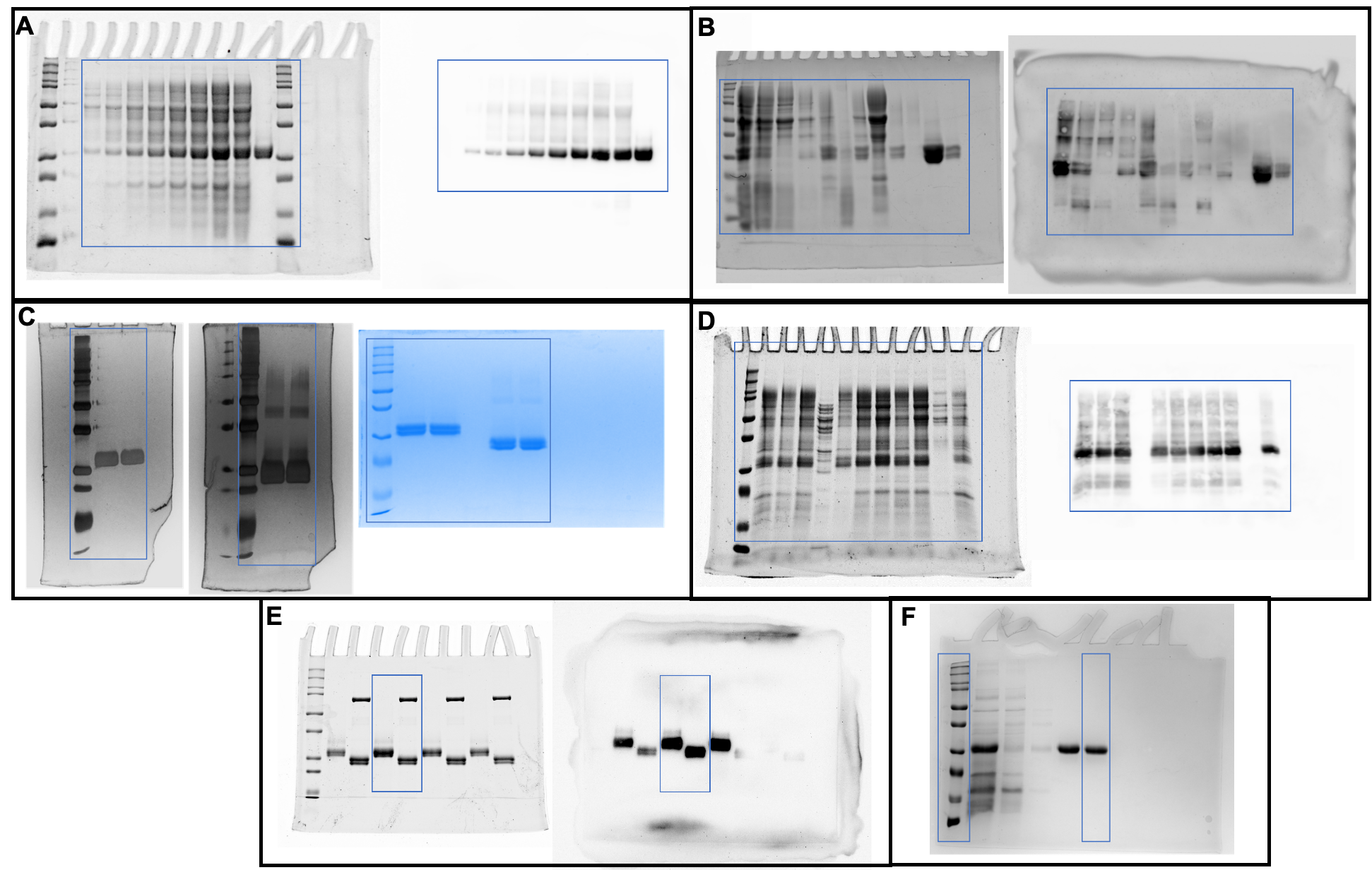


**Supplementary Information.** Full images of SDS-PAGE and/or western blots for **(A)** Figure 1C, **(B)** Figure 2A, **(C)** Figure 3B, 3C, **(D)** Supplementary Figure 1A, **(E)** Supplementary Figure 3A, **(F)** Supplementary Figure 3E.
