# Supplementary material for: Process development for an effective COVID-19 vaccine candidate harboring recombinant SARS-CoV-2 delta plus receptor binding domain produced by Pichia pastoris
Source: Sci Rep. 2023 Mar 30;13:5224. doi: 10.1038/s41598-023-32021-9 (PMC10062263; doi:10.1038/s41598-023-32021-9)
Supplement: Supplementary file 2 — Supplementary Information 2. [file 41598_2023_32021_MOESM2_ESM.docx]

**Process development for an effective COVID-19 vaccine candidate harboring recombinant SARS-CoV-2 Delta Plus receptor binding domain produced by *Pichia pastoris***

Sibel Kalyoncu^1^, Semiramis Yilmaz^1,2^, Ayca Zeybek Kuyucu^1^, Dogu Sayili^1^, Olcay Mert^1^, Hakan Soyturk^1^, Seyda Gullu^1^, Huseyin Akinturk^1^, Erhan Citak^1,2^, Merve Arslan^1,3^, Melda Guray Taskinarda^1^, Ibrahim Oguzhan Tarman^1^, Gizem Yilmazer Altun^1^, Ceren Ozer^1,3^, Ridvan Orkut^1^, Aysegul Demirtas^1^, Idil Tilmensagir^1^, Umur Keles^1,4^, Ceren Ulker^1^, Gizem Aralan^1^, Yavuz Mercan^1,3^, Muge Ozkan^1^, Hasan Onur Caglar^1,5^, Gizem Arik^1,6^, Mehmet Can Ucar^1,7^, Muzaffer Yildirim^1^, Tugce Canavar Yildirim^1^, Dilara Karadag^1^, Erhan Bal^1,8^, Aybike Erdogan^1,3^, Serif Senturk^1,3^, Serdar Uzar^9^, Hakan Enul^9^, Cumhur Adiay^9^, Fahriye Sarac^9^, Arzu Tas Ekiz^10^, Irem Abaci^10^, Ozge Aksoy^10^, Hivda Ulbegi Polat^10^, Saban Tekin^10,11^, Stefan Dimitrov^1^, Aykut Ozkul^12^, Gerhard Wingender^1^, Ihsan Gursel^1^, Mehmet Ozturk^1,8^, Mehmet Inan^1,13,*^

^1^ Izmir Biomedicine and Genome Center, Izmir, Turkey,

^2^ Current address: VIB-UGent Center for Medical Biotechnology, Gent, Belgium

^3^ Dokuz Eylul University, Izmir International Biomedicine and Genome Institute, Izmir, Turkey

^4^ Current address: Lund University, Lund, Sweden

^5^ Current address: Erzurum Technical University, Erzurum, Turkey

^6^ Current address: Ankara Medipol University, Ankara, Turkey

^7^ Current address: Imperial College London, London, United Kingdom

^8^ Current address: Izmir Tinaztepe University, Izmir, Turkey

^9^ Pendik Veterinary Research and Control Institute, Istanbul, Turkey

^10^ Marmara Research Center, TUBITAK, Kocaeli, Turkey

^11^ University of Health Sciences, Istanbul, Turkey

^12^ Ankara University, Ankara, Turkey

^13^ Akdeniz University, Antalya, Turkey

*Corresponding author: Mehmet Inan, E-mail: mehmet.inan@ibg.edu.tr

**Keywords:** SARS-CoV-2, Delta strain, COVID-19 vaccine, Pichia Pastoris, recombinant subunit vaccine.

**Supplementary Tables and Figures**

**Supplementary Table 1.** Protein purification table for RDB-DP.

| **Process Steps** | **Volume(~mL)- Concentration (~mg/mL)** | **Amount (mg)** | **Purity**  **(%)** | **Step recovery**  **(%)** |
| --- | --- | --- | --- | --- |
| Harvest-Supernatant | 100 – 1.20 | 120.0 | 15 ± 3 | 100 |
| Filtration | 100 – 1.06 | 105.6 | 17 ± 3 | 88 ± 2 |
| HIC (Capture) | 65 – 1.19 | 77.1 | 70 ± 5 | 73 ± 3 |
| Desalting-Buffer Exchange | 90 – 0.85 | 76.3 | 70 ± 5 | 99 ± 1 |
| AEX (Intermediate) | 93 – 0.60 | 55.7 | 90 ± 2 | 73 ± 2 |
| CEX (Polishing) | 12 – 2.55 | 30.6 | 96 ± 1 | 55 ± 2 |
| Buffer Exchange and Storage | 15 – 2.00 | 30.0 | 96 ± 1 | 98 ± 1 |
| **Overall** | **15 – 2.00** | **30.0** | **96 ± 1** | **25 ± 3** |


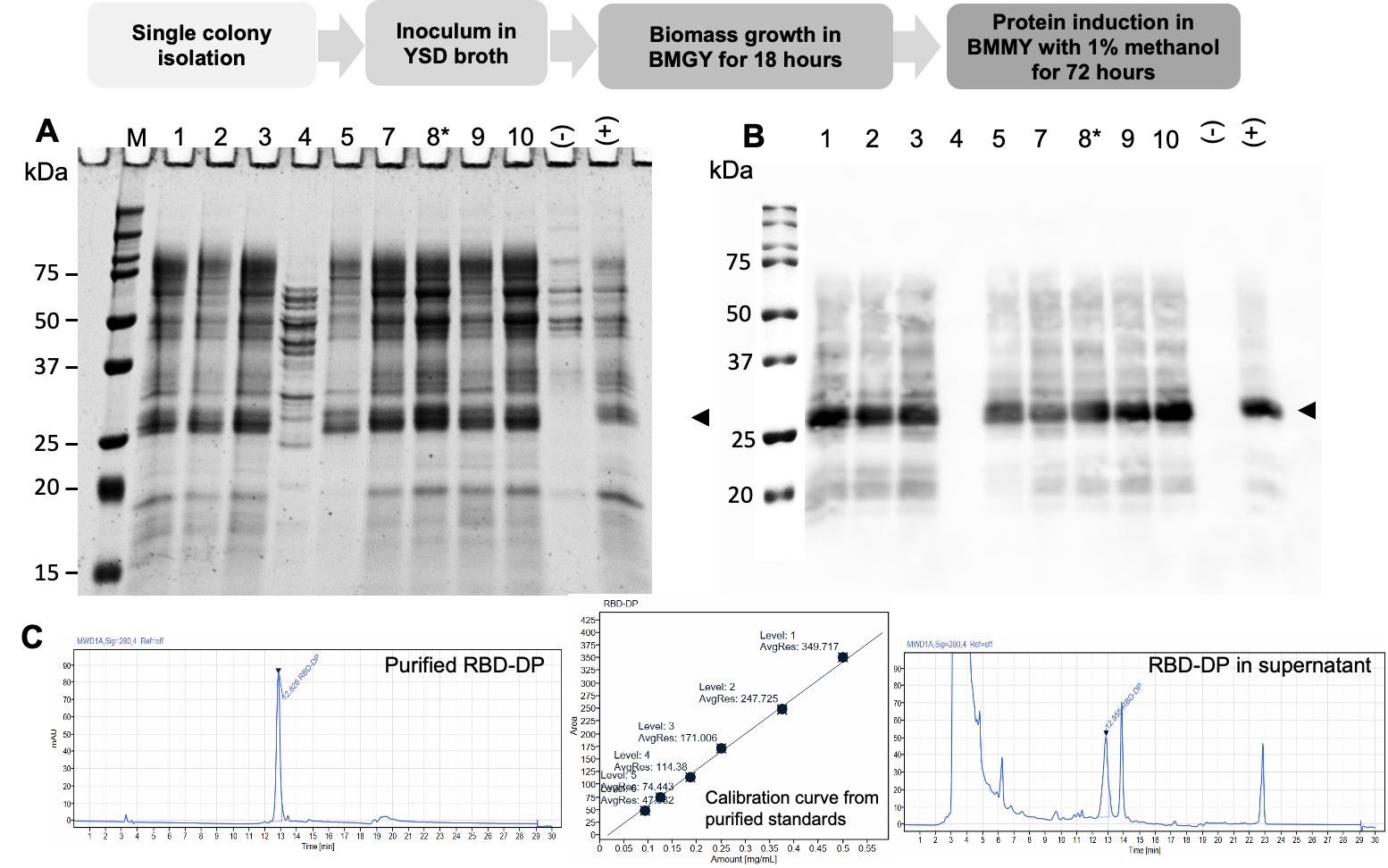


**Supplementary Figure 1.** Screening of the transformant clones to select the best RBD-DP protein expressing one. **(A)** SDS-PAGE and **(B)** Western blot analysis. M: DNA Ladder, (1-10) The clone numbers of clones selected from transformation plates to be isolated as single colony, (-) Untransformed host cell, (+) RBD-UK variant expression clone, (*) selected as best expression (production) clone. **(C)** Left pane: RP-HPLC chromatogram of purified RBD-DP protein (2 mg/mL), middle pane: standard calibration curve generated by OpenLab CDS from purified RBD-DP proteins with double injection, right pane: RP-HPLC chromatogram of fermentation supernatant. The peak corresponding to RBD-DP was labelled at 12.8 min retention time.


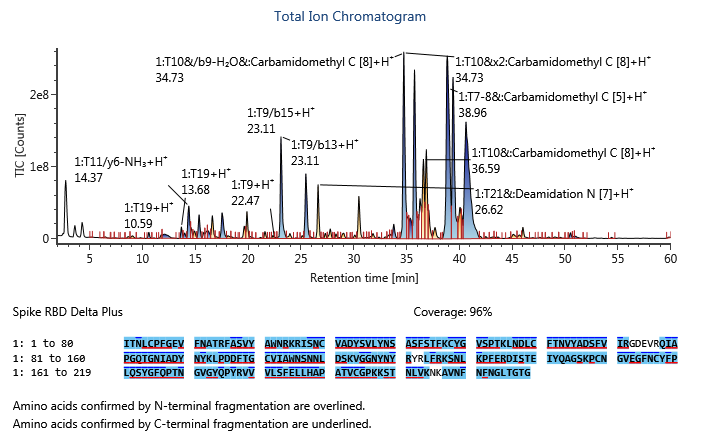


**Supplementary Figure 2.** Total ion chromatogram (top) and sequence coverage map (bottom) of RBD-DP obtained by peptide mapping analysis.


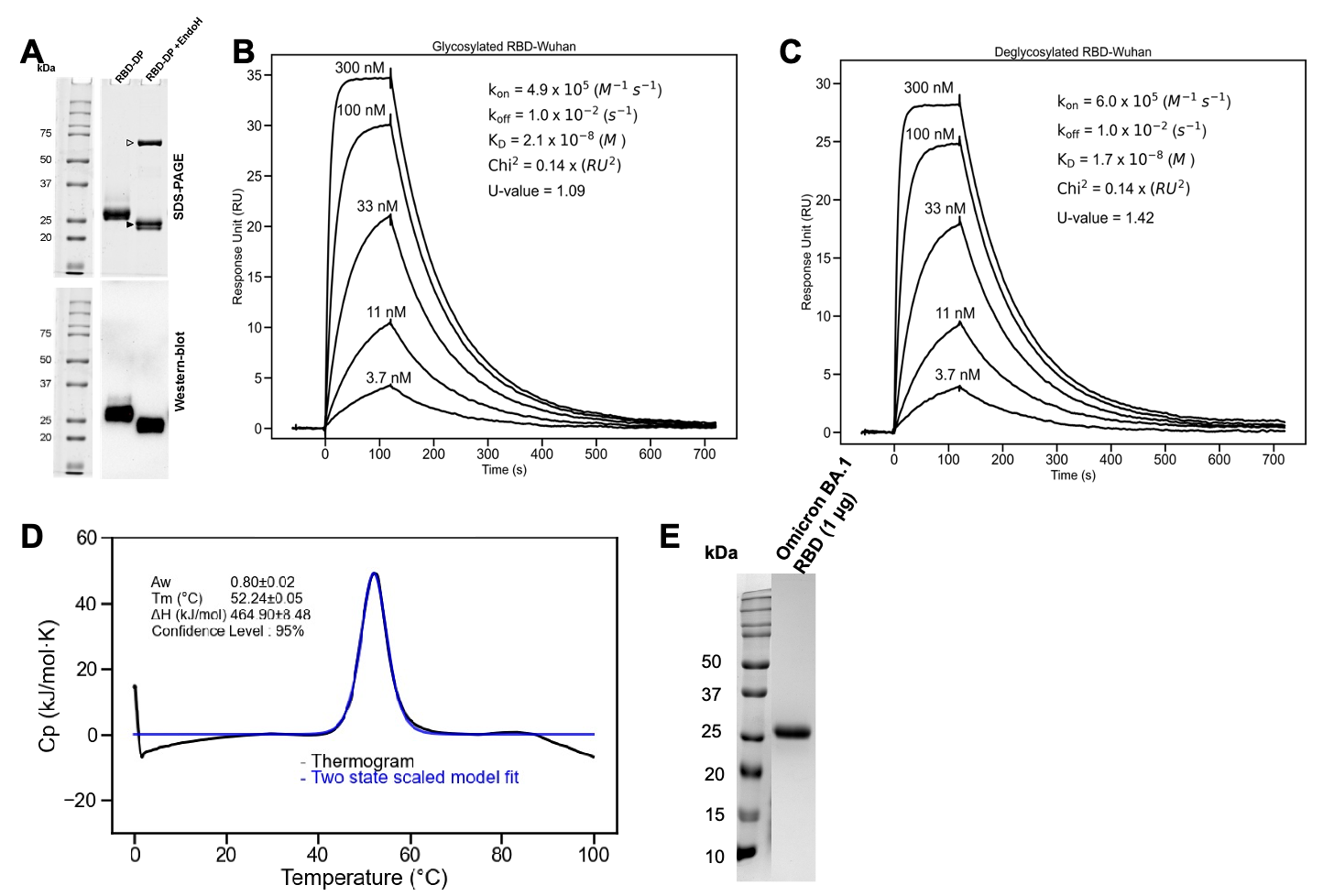


**Supplementary Figure 3.** Analysis of deglycosylated RBD-DP. **(A)** SDS-PAGE and Western blot analysis of RBD-DP protein (4 μg) before and after deglycosylation by EndoH treatment. Apparent molecular weight of EndoH is 70 kDa on the gel. ACE2-Fc SPR binding sensogram of **(B)** glycosylated and **(C)** deglycosylated RBD-Wuhan, indicating their similar binding kinetics. **(D)** Differential Scanning Calorimetry (DSC) profile of WT-RBD showing thermal melt of 52.24 ± 0.10 ºC. **(E)** SDS-PAGE image of produced and purified Omicron BA.1 RBD domain. This protein was used for ELISA in Figure 4E.


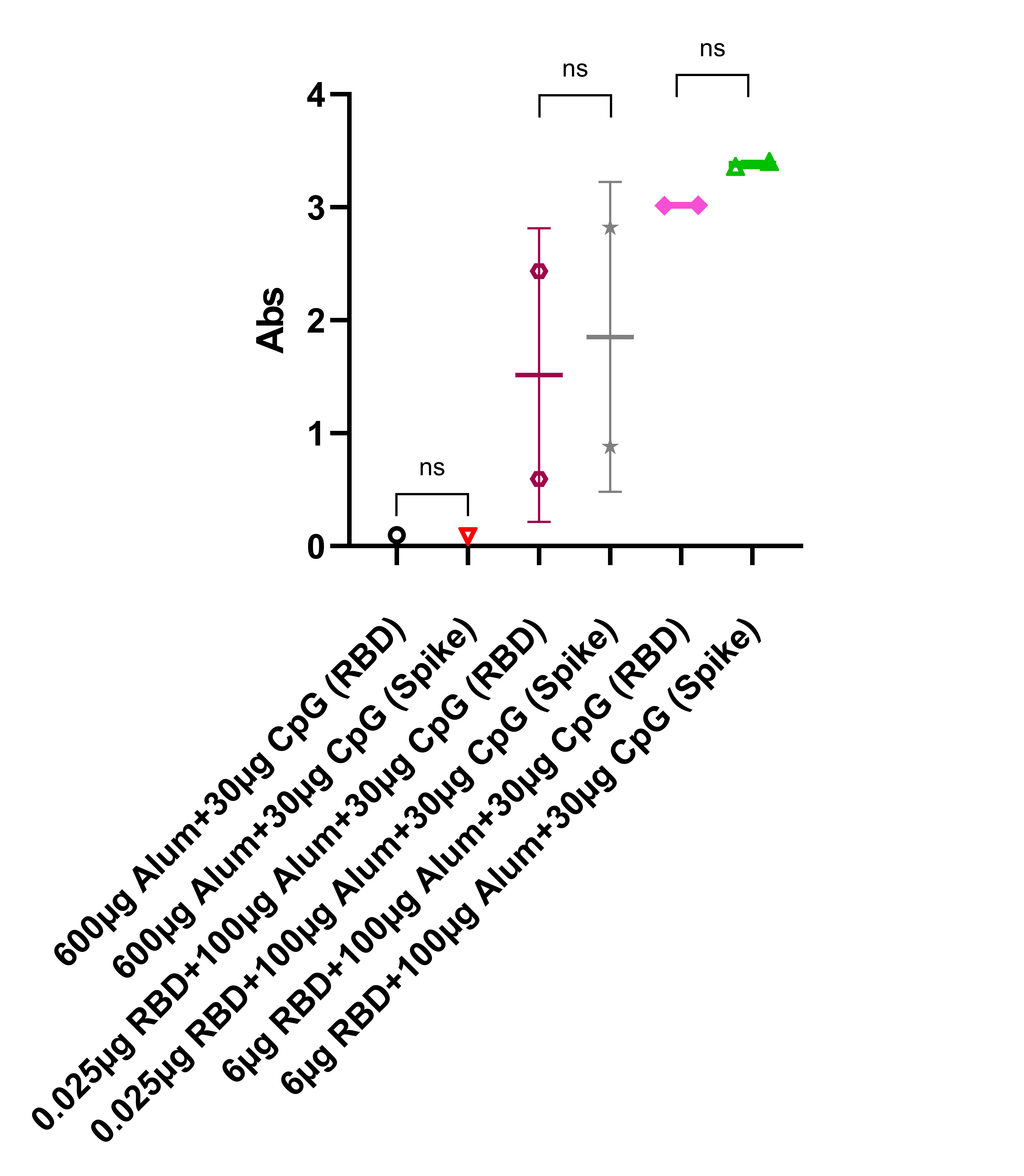


**Supplementary Figure 4.** ELISA analysis of sera from animals immunized with different doses of RBD-DP: 1 animal of control (Alum+CpG), 2 animals of low dose (0.025 μg RBD-DP) and 2 animals of high dose (6 μg RBD-DP). ELISA plates were coated with either RBD-DP (RBD) or full-length WT Spike protein (Spike). The difference between absorbance values of RBD-DP and Spike coated wells were not significant according to one-way ANOVA-Tukey’s multiple comparisons test (ns: not significant).


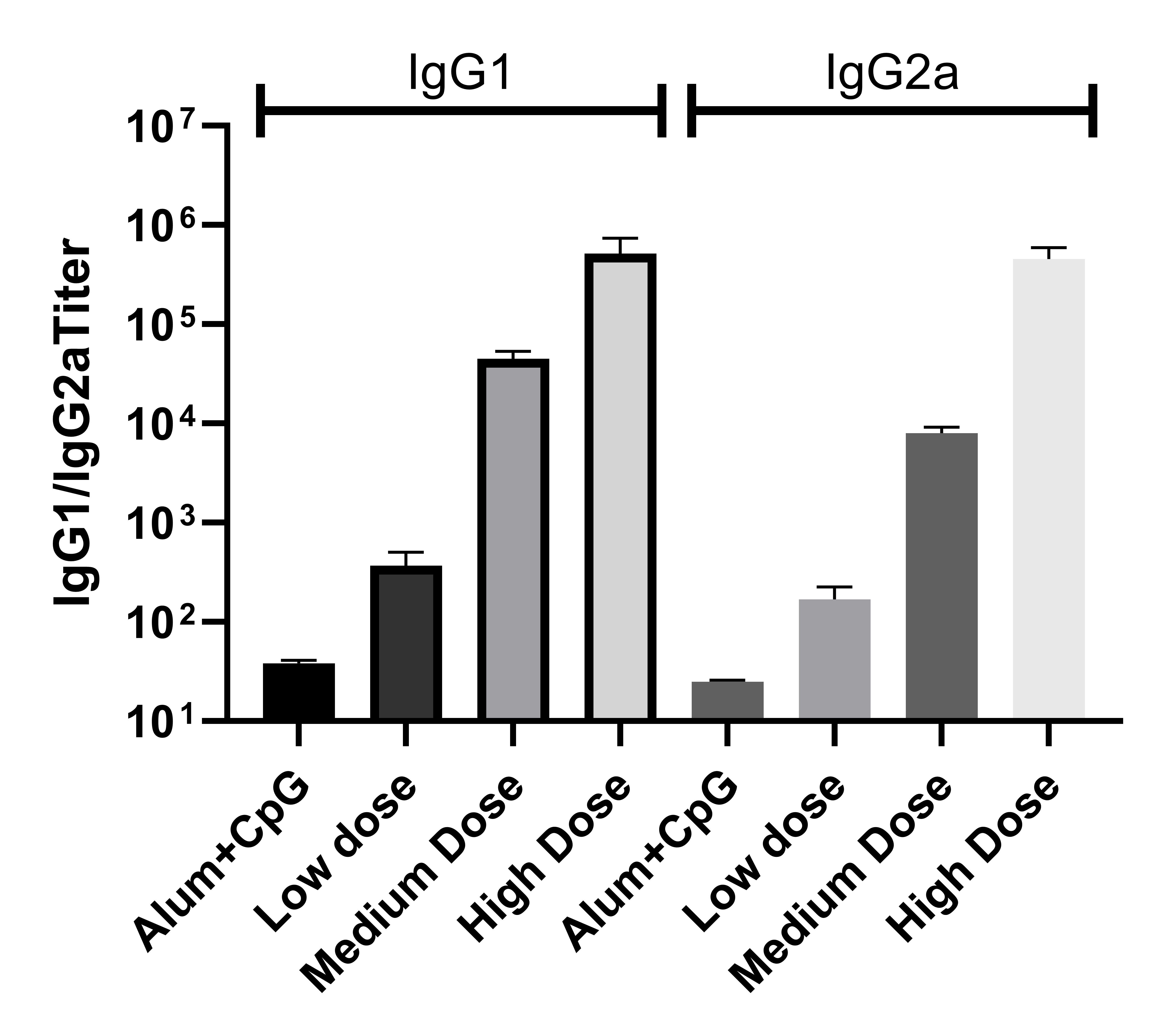


**Supplementary Figure 5.** Titers of IgG1 and IgG2a subtypes in mice after immunized twice with three different doses (low dose: 1 µg, medium dose: 5 µg, high dose: 25 µg RBD-DP with 60 µg Alum, 30 µg CpG for each).


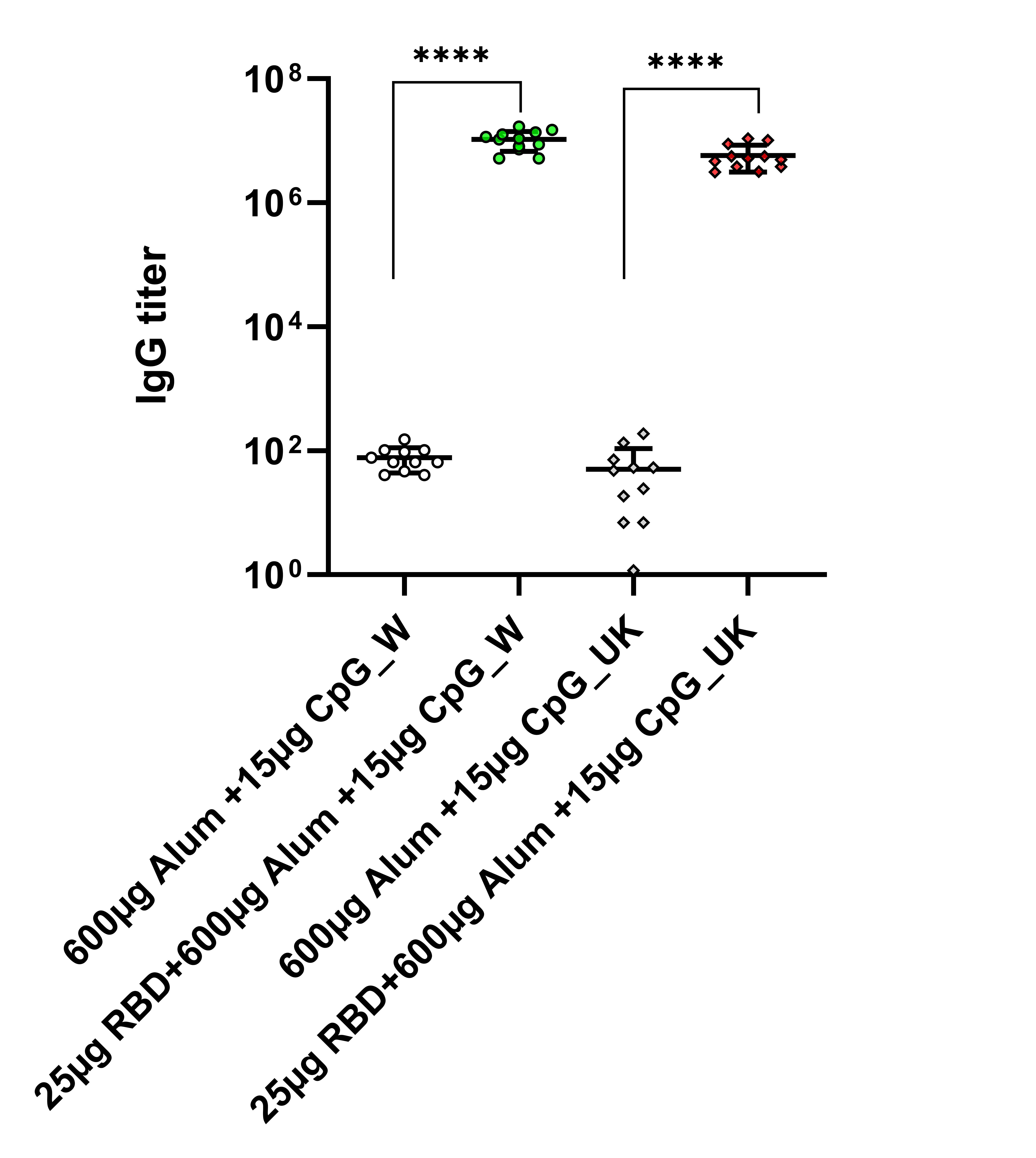


**Supplementary Figure 6.** Total IgG titers in mice after immunized 3-doses with RBDs of Wuhan (W) and UK variants at day 49 (please refer to Figure 4B). Formulation information was embedded into the figure. Groups were compared by one-way ANOVA-Tukey’s multiple comparisons test (****: p<0.0001).
